# Transplacental passage of hyperforin, hypericin, and valerenic acid

**DOI:** 10.3389/fphar.2023.1123194

**Published:** 2023-03-31

**Authors:** Deborah Spiess, Vanessa Fabienne Abegg, Antoine Chauveau, Joshua Rath, Andrea Treyer, Michael Reinehr, Sabrina Kuoni, Mouhssin Oufir, Olivier Potterat, Matthias Hamburger, Ana Paula Simões-Wüst

**Affiliations:** ^1^ Department of Obstetrics, University Hospital Zurich, University of Zurich, Zurich, Switzerland; ^2^ Division of Pharmaceutical Biology, Department of Pharmaceutical Sciences, University of Basel, Basel, Switzerland; ^3^ Department of Pathology and Molecular Pathology, University Hospital Zurich, Zurich, Switzerland

**Keywords:** hyperforin, hypericin, valerenic acid, *Hypericum perforatum*, *Valeriana officinalis*, placental perfusion, pregnancy, U(H)PLC-MS/MS

## Abstract

Safe medications for mild mental diseases in pregnancy are needed. Phytomedicines from St. John’s wort and valerian are valid candidates, but safety data in pregnancy are lacking. The transplacental transport of hyperforin and hypericin (from St. John’s wort), and valerenic acid (from valerian) was evaluated using the *ex vivo* cotyledon perfusion model (4 h perfusions, term placentae) and, in part, the *in vitro* Transwell assay with BeWo b30 cells. Antipyrine was used for comparison in both models. U(H)PLC-MS/MS bioanalytical methods were developed to quantify the compounds. Perfusion data obtained with term placentae showed that only minor amounts of hyperforin passed into the fetal circuit, while hypericin did not cross the placental barrier and valerenic acid equilibrated between the maternal and fetal compartments. None of the investigated compounds affected metabolic, functional, and histopathological parameters of the placenta during the perfusion experiments. Data from the Transwell model suggested that valerenic acid does not cross the placental cell layer. Taken together, our data suggest that throughout the pregnancy the potential fetal exposure to hypericin and hyperforin – but not to valerenic acid – is likely to be minimal.

## Introduction

Pregnancy is a vulnerable period for mental disorders and/or symptoms. Studies from various countries (e.g., low-middle-income countries, United States, Sweden), reported a prevalence of 14%–20% for psychiatric diseases in pregnancy (Andersson et al., 2003; Marcus et al., 2003; Fisher et al., 2012). In Switzerland, the annual rate of perinatal women using mental health services accounted for 16.7% (Berger et al., 2017). Moreover, a Swiss cross-sectional survey revealed that more than 52.0% of the participants suffered from mental disorders and/or symptoms during pregnancy but only a few (1.6%) took synthetic psychoactive medications (Gantner et al., 2021). Mental disorders like mild depression, sleep disorders, and anxiety can lead to complications like preterm birth if left untreated (Dunkel Schetter, 2011). However, most synthetic drugs may not only cause side effects in the mother, but also cross the placental barrier and reach the fetus. Concerns on tolerability, teratogenicity and impact on neonatal outcomes exist and are, in part, supported by various studies (Sivojelezova et al., 2005; Rahimi et al., 2006; Grigoriadis et al., 2014; Yonkers et al., 2014; Gao et al., 2018). Pregnant women in need of medications such as selective serotonin reuptake inhibitors (SSRIs) and benzodiazepines therefore face the dilemma of either using or refraining from using them.

Phytomedicines are popular alternatives to synthetic medications. Many pregnant women use herbal medicines, in addition to or rather than synthetic drugs, probably as they perceive those alternatives to be the safer choice for their unborn child (Sarecka-Hujar and Szulc-Musiol, 2022). A recent Swiss survey revealed that 89.9% of pregnant women are using some type of herbal medicine (Gantner et al., 2021). Some healthcare professionals also tend to recommend phytomedicines and, hence, contribute to the trust in these products (Stewart et al., 2014). Most phytomedicines are available without prescription. In general, safety data for use during pregnancy are lacking for phytomedicines (Bernstein et al., 2021; Morehead and McInnis, 2021). For example, it is not known whether pharmacologically active compounds in these products can cross the placental barrier. Given the lack of data, the agencies responsible for approval of drugs require a warning label in the patient information of these products.

In the treatment of mild to moderate depression, St. John’s wort (*Hypericum perforatum* L., Hypericaceae) is an alternative to SSRIs, as the clinical efficacy has been documented in several clinical trials (Linde et al., 2008). Hyperforin and hypericin are two characteristic compounds in St. John’s wort. They have been shown to possess various CNS-related pharmacological activities but are not solely responsible for the antidepressant properties. As for most other phytomedicines, the entire extract has to be considered as the active ingredient (Butterweck and Schmidt, 2007; Nicolussi et al., 2020). A recent study based on data from Germany found that pregnant women mainly used St. John’s wort in the first trimester, but simultaneous dispensation of other drugs that favour interactions and the observation of a relatively high rate of non-live births call for a thorough further safety investigation (Schäfer et al., 2021). Valerian *(Valeriana officinalis* L., Caprifoliaceae) is known for its sleep promoting and anxiolytic properties (Hattesohl et al., 2008). Valerenic acid, a characteristic compound in valerian, is an allosteric modulator of GABA_A_ receptors (Becker et al., 2014), but again is not solely responsible for the clinical efficacy of phytomedicines containing valerian extracts. Valerian is favoured by many expecting mothers, as shown in a multinational study where valerian was among the five most frequently used herbal medicines (Kennedy et al., 2013). St. John’s wort and valerian have been used for decades in Europe and have been labelled with “well established use” and “traditional use”, respectively, by the Committee on Herbal Medicinal Products (HMPC) of the European Medicines Agency (EMA). However, they recommend neither of the herbs to be used during pregnancy due to insufficient toxicological data (European Medicines Agency, 2015; European Medicines Agency, 2021).

Relevant toxicological aspects in the context of pregnancy include effects on placental function and on transplacental transfer. The human placenta develops during pregnancy, at each gestational stage supplying the developing fetus with blood, nutrients, and oxygen, while also regulating the removal of waste products and carbon dioxide. In addition, it metabolises substances and releases hormones that influence the course of pregnancy, fetal metabolism and growth, and labour itself (Gude et al., 2004). The placenta also protects the fetus from infections, maternal diseases, and some xenobiotics including drugs. Most drugs pass through the placenta *via* passive or simple diffusion that is influenced by factors such as molecular weight (MW), degree of ionization, lipid solubility, protein binding, concentration gradient of the drug across the placenta, placental surface area/thickness, pH of maternal and fetal blood, and placental metabolism (van der Aa et al., 1998; Syme et al., 2004; Tetro et al., 2018). Other processes of drug transfer across the placenta include facilitated diffusion, active transport, and endocytosis. Once formed, the placental syncytiotrophoblast is the rate-limiting barrier separating the maternal and fetal circulation, with various transporters and enzymes located at the apical and basolateral membranes (Desforges and Sibley, 2010; Al-Enazy et al., 2017). Prior to their formation, a monolayer of precursor cells (cytotrophoblasts) held together by tight junctions exerts the barrier function (Prouillac and Lecoeur, 2010). Data on transplacental passage of drugs can be obtained using the *ex vivo* human placental perfusion model (with term placentae) representative of the late stage of pregnancy (Myllynen and Vahakangas, 2013). It is considered to be the gold-standard among placental transfer models (Panigel et al., 1967; Malek et al., 2009; Grafmuller et al., 2013), and we have recently shown its usefulness for studying the transplacental transfer of phytochemicals (Spiess et al., 2022a). *In vitro* Transwell models utilising monolayers of confluent, human, non-differentiated placental cells, on the other hand, reflect the transfer through a continuous layer of cytotrophoblasts (Bode et al., 2006; Vähäkangas and Myllynen, 2006; Prouillac and Lecoeur, 2010).

**FIGURE 1:**
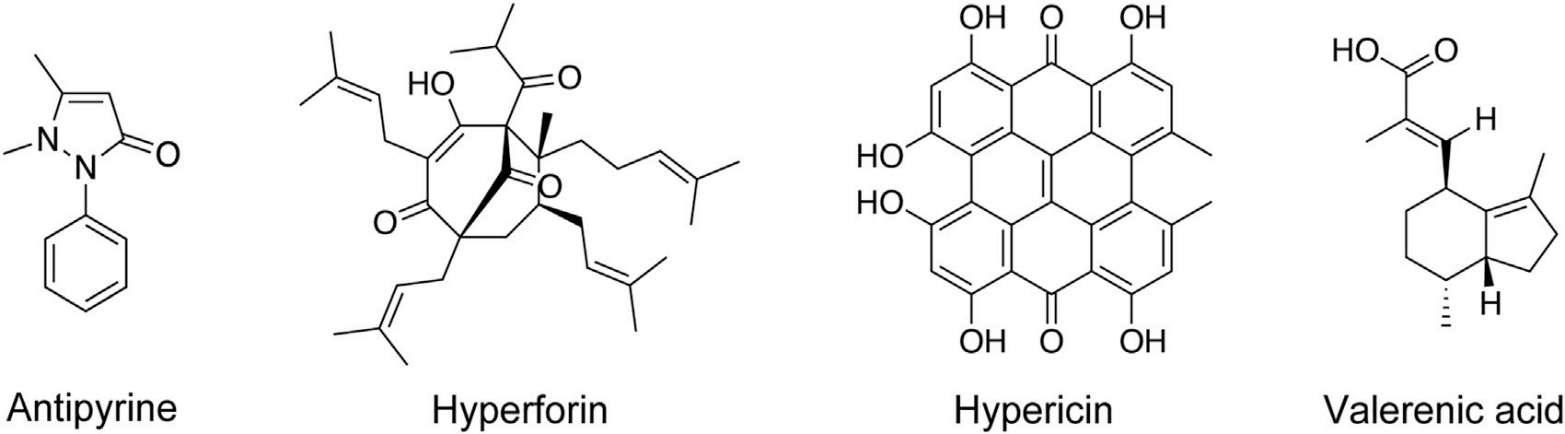
Chemical structures of investigated phytochemicals and antipyrine.

In the present study, we investigated the transplacental passage of hyperforin, hypericin, and valerenic acid (Figure 1) using the *ex vivo* term placental perfusion model. In this model, we also investigated their effects on metabolic, functional, and histopathological properties of placental tissue. In case of valerenic acid, an *in vitro* Transwell model based on the human placental BeWo b30 cell line (Li et al., 2013) was used in addition.

## Materials and methods

### Chemicals, reagents, and study compounds

All solvents were of UPLC grade. Acetonitrile (MeCN) was purchased from Merck. Methanol was from Avantor Performance Materials Poland. Purified water was obtained from a Milli-Q integral water purification system. Dimethyl sulfoxide (DMSO) was supplied by Scharlau, and formic acid was from BioSolve. Antipyrine, hyperforin dicyclohexylammonium salt and bovine serum albumin (BSA) were obtained from Sigma-Aldrich, and antipyrine-d_3_ from HPC Standards. Valerenic acid was purchased from Extrasynthese and PhytoLab, and hypericin was from Carbosynth. Warfarin was purchased from Toronto Research Chemicals, and digoxin from Sigma-Aldrich.

### *Ex vivo* human placental perfusion

#### Placentae collection

Placentae were collected with informed written consent from women with uncomplicated term pregnancies immediately after undergoing primary caesarean section. This procedure was approved by the Ethics Committee of the Canton of Zurich (KEK-StV73 Nr. 07/07; 21 March 2007). All research was performed in accordance with the Declaration of Helsinki and other relevant guidelines/regulations. All placentae were verified to be negative for HIV, HBV, SARS-CoV-2, and twin pregnancy donors were not included in the study. Placentae with a ragged maternal surface (visible disruptions; macroscopic tissue trauma), evidence of basal plate fibrin deposition, suspected placental infarction or too little fetal membrane (on the disk of the placenta) were not considered for perfusion. Supplementary Table S2 gives an overview of the experimental conditions and characteristics of all placentae used: a total of 11 placentae (donated by 11 women) were suitable for perfusion, 3-4 individual placentae were used for each test substance.

#### Equipment and experimental procedure of perfusion

The *ex vivo* human placental perfusion model was adapted from the models of Schneider (Schneider, 1972) and Grafmüller (Grafmuller et al., 2013), and has been described in detail (Spiess et al., 2022a). In brief, one villous tree of a cotyledon (placenta lobule) was perfused by cannulation of the chorionic artery and corresponding vein. Three blunt cannulae were inserted into the intervillous space to reconstruct the maternal circuit and to allow the transplacental exchange through the perfusion medium (PM). The PM consisted of Earle’s buffer (1 part), cell culture medium 199 (2 parts; Sigma-Aldrich), and supplements (BSA, dextran, glucose, sodium bicarbonate, amoxicillin, and heparin). The fetal perfusate was gassed with 95% N_2_/5% CO_2_, and the maternal perfusate with 95% air/5% CO_2_ instead. Two heating magnetic stirrers ensured a constant distribution of study compounds in both reservoirs. A physiological temperature of 37°C was maintained with flow heaters (heating columns) and a water bath. Digitally controlled peristaltic pumps (Ismatec) transported the fetal and maternal perfusates at a rate of 3 and 12 mL/min, respectively. The perfusion experiment, including a 20 min non-recirculating (open) and a 20 min recirculating (closed) preparatory phase, started with equal volumes of 100 mL fresh PM. The study compound and antipyrine were added to the perfusate of the maternal circuit, both at a final concentration of 5 μM (≙ 941 ng/mL antipyrine, 2′684 ng/mL hyperforin, 2′522 ng/mL hypericin, and 1′172 ng/mL valerenic acid).

The dissolution and adherence of the compounds to the perfusion equipment and to the tubing system was assessed before starting perfusions with human placentae. All study compounds were pumped over a period of 240 min through an empty (i.e., in the absence of placental tissue) perfusion chamber comprising only the maternal circuit. The compounds were directly dissolved in PM at a final concentration of 5 μM (100 mL reservoir) and individually tested in three independent experiments (n = 3).

#### Sample preparation and quality controls

Antipyrine served as connectivity (positive) control in all placental perfusions to verify the overlap of the maternal and the fetal circulation. The stability of volumes in each reservoir ensured the integrity of the circuits and served for the detection of fetal-maternal (FM) leaks (≤4 mL/h). Throughout the perfusion additional quality control measures included a physiological pH-range (7.2 ± 0.1) and a controlled fetal perfusion pressure (≤ 70 mmHg). Samples were taken at defined timepoints over a 240 min period, immediately centrifuged, and stored in glass micro-inserts (VWR) at −80°C for bioanalytical analysis. A blood gas analysis (pH, *p*O_2_, *p*CO_2_, glucose, lactate; ABL800 FLEX) of fetal and maternal samples was performed to ensure viability and metabolic activity of placental tissue during perfusion. The production of the placental hormones beta-human chorionic gonadotropin (*β*-hCG) and leptin was monitored by standard ELISA to assess tissue functionality *ex vivo* [see (Spiess et al., 2022a) and references therein]. For *β*-hCG, flat-bottom microplates were coated with rabbit polyclonal anti-hCG antibody (Agilent Dako) at a 1:1′000 dilution and the mouse monoclonal anti-hCG (abcam; 1:5′000) served as secondary antibody. The peptide hormone hCG (Lucerna-Chem) was used as reference standard; standard final concentrations of between 100 mU/mL and 2.5 mU/mL were prepared by serial dilution in seven steps and used in each plate. The goat anti-mouse-IgG-horseradish peroxidase conjugate (abcam) antibody was used at a 1:5′000 dilution. The substrate consisted of O-phenylenediamine dihydrochloride. Intra-assay CV% was ≤10%, inter-assay CV% was <6% at 100 mU/mL and <15% at 10 mU/mL. For leptin, microtiter plates were coated with mouse monoclonal anti-human leptin/OB (R&D Systems; 1:250). The second antibody was biotinylated monoclonal mouse anti-human leptin/OB (R&D Systems), used at a dilution of 1:1′000. The standard was recombinant human leptin (R&D Systems); standard final concentrations of between 2′000 pg/mL and 15.6 pg/mL were prepared by serial 1:2 dilution in seven steps and used in each plate. Intra-assay CV% was ≤10%, inter-assay CV% was <3% at 2′000 pg/mL and <15% at 250 pg/mL. The conjugate streptavidin-horseradish peroxidase (Southern Biotechnology Associates, 1:4′000) was added and the plate incubated for 60 min. For the development, ready-to-use tetramethylbenzidine substrate solution (Thermo Fischer) was used.

#### Histopathological evaluation

Immediately after perfusion the placentae were fixed in 4% paraformaldehyde for at least 24 h. Tissue samples from representative placental tissue sections, each from perfused, non-perfused, and transitional area were prepared to perform a pathological examination. For this purpose, the tissue was embedded in paraffin, cut (∅ 2-3 µm), and stained (standard hematoxylin-eosin stain, Braun-Brenn modified Gram stain) according to the standards of routine histopathological diagnosis of the Department of Pathology and Molecular Pathology (University Hospital of Zurich). The tissue of the non-perfused specimens was examined with regard to general placental pathologies as described in routine diagnostics (Turowski et al., 2012; Khong et al., 2016). The blood void and width of the intervillous space and fetal blood vessels in the chorionic villi provided information about the quality of perfusion, and particular attention was paid to the presence of intravascular thrombi. General signs of degeneration, such as vacuolization of the cytotrophoblast, villous vascular endothelium viability, and formation of hydropic villous changes compared with non-perfused tissue indicated whether tissue damage might have occurred during perfusion. Microscopic effects and placental tissue damage in the perfused area were expressed in relative amounts (%) to the non-perfused tissue.

### *In vitro* permeability assay

#### Cell culture

BeWo b30 choriocarcinoma cells were obtained from Dr. Tina Buerki-Thurnherr (Empa, St. Gallen, Switzerland), with permission from Dr. Alan L. Schwartz (Washington University School of Medicine, St. Louis MO, USA), and were cultivated in F-12 K Nut Mix supplemented with 10% heat inactivated FBS, antibiotics (100 U/mL penicillin, 100 µg/mL streptavidin), and 2 mM L-glutamine (all from Gibco). The cells were cultivated in a humidified incubator at 37°C and 5% CO_2_ atmosphere.

#### Monolayer formation on cell culture inserts

BeWo b30 cells were cultured on Transwell^®^ polycarbonate membrane inserts (24-well format; 0.4 μm pore size, 0.33 cm^2^ cell growth area, 200 μL apical volume, 1′000 μL basolateral volume; Corning, Sigma-Aldrich) at a density of 60′000 cells/well. These inserts were then cultivated in a cellZscope (nanoAnalytics) at 37°C/5% CO_2_. Cell culture medium was replaced every 2 days up to 11 days to find the best possible conditions.

#### Evaluation of monolayer formation

Measurement of transepithelial electrical resistance (TER) was used to assess the tightness of a cell-to-cell barrier, and the electrical capacitance C_cl_, provided additional information about the properties of the cell layer (e.g., presence of microvilli and other membrane extrusions). TER and C_cl_ values were recorded in 15 min time intervals, using a cellZscope^®^. TER values were corrected for the surface area (Ωcm^2^) and the reference resistance (well with the same filter insert and medium, but absence of cells). Moreover, a permeability assay with sodium fluorescein (NaF) was performed on days 7–11 (n = 3). The basolateral compartment of a transparent 24-well plate consisted of PBS only (1′000 μL) while NaF (5 μM in PBS; 200 μL) was added to the apical compartment for 60 min. The control consisted of cell-free inserts (n = 3). Basolateral samples (50 μL) were directly added to black Nunc MaxiSorp microtiter plates, and concentrations were determined using a Cytation 3 fluorescence microplate reader (BioTek Instruments; excitation wavelength 460 nm; emission wavelength 515 nm).

#### Immunocytochemical staining of cells on inserts

BeWo b30 cells were stained with fluorescent probes for nuclei and cytoplasm as follows. Cells were fixed in 4% paraformaldehyde (Artechemis) for 20–30 min. Afterwards they were permeabilized and blocked with 0.3% Triton-X-100 (Sigma) in 1% BSA (Thermo Scientific) in PBS (Gibco) for another 20–30 min at room temperature on a shaker (50 rpm). Then, cells were incubated at room temperature on a shaker (50 rpm) for 4–6 h, wrapped in tinfoil, with a 1:10′000 solution of 4′,6-diamidino-2-phenylindole (DAPI; Sigma) and a 1:400 phalloidin-rhodamine (Invitrogen) solution diluted with 0.1% Triton X-100 in 1% BSA/PBS. Cells were then extensively washed with PBS, and the insert membranes were embedded between glass cover slides using Mowiol 4–88 (Sigma-Aldrich) to obtain flat membranes. The images were acquired with a Leica CTR 6000 microscope (Leica Microsystems) and the corresponding Leica Application Software X.

#### Permeability assay

BeWo b30 cells were cultured in transparent 24-well plates under the same conditions as mentioned above and transferred to the cellZscope^®^ at day 8 to record TER and C_cl_ values. After 24 h (day 9) and when a TER of 30–60 Ωcm^2^ was reached and C_cl_ was between 0.5 and 5.0 μF/cm^2^, the permeability assay was initiated by adding valerenic acid (5 μM; in HBSS with 4% BSA) to the apical compartment, while the basolateral compartment contained 1′000 μL of HBSS only. In addition, antipyrine (5 μM) was added along with the test substance as a control. Samples (150 μL apical, 800 μL basolateral) were collected at each time point (0, 15, 30, 60 min; one insert per time point). After 60 min, a part of the inserts was transferred back to the cellZscope^®^ to monitor TER and C_cl_ values during another 24 h. The cells grown on the other part of the inserts were quickly washed with 200 μL of cold HBSS and then lysed with 700 μL acetonitrile (100 μL apical, 600 μL basolateral) for 40 min on an orbital shaker (450 rpm, room temperature) to determine the test substance cell contents.

#### Calculation of permeability coefficients and recovery

Apparent permeability coefficients (P_app_) were calculated according to the following Eq. 1:

$$P_{app}\;\left(cm/s\right)=\frac{∆Q/∆t}{AC_{A0}}$$ (1)

where ΔQ/Δt is the rate of amount transported to the receiver compartment, *A* is the membrane surface area (0.33 cm^2^), and *C*_*A0*_ is the initial concentration in the apical compartment.

The clearance values were calculated according to the following Eq. 2 (Neuhaus et al., 2008):

$$Clearance\;\left(\mu L\right)=\frac{C_{Bn}V_{B}}{C_{A}-\left(\frac{V_{B}}{V_{A}}∙\sum C_{Bn-1}\right)}$$ (2)

where *C*_*Bn*_ and *V*_*B*_ are the concentration and volume in the basolateral compartment at a specific timepoint (n), respectively; *C*_*A*_ and V_A_ are the concentration and volume in the apical compartment, respectively, and *C*_*Bn-1*_ is the total amount of substance found in the basolateral compartment up to the previous timepoint (n-1).

Recovery (mass balance) of each compound was calculated according to the following Eq. 3:

$$Recovery\;\left(\%\right)=\frac{C_{Af}V_{A}+C_{Bf}V_{B}+C_{Cf}V_{C}}{C_{A0}V_{A}}∙100$$ (3)

where *C*_*Af*_, *C*_*Bf*_ and *C*_*Cf*_ are the final compound concentrations in apical, basolateral, and cellular compartments, respectively; *C*_*A0*_ is the initial concentration in the apical compartment, and *V*_*A*_, *V*_*B*_ and *V*_*C*_ are the volumes in the respective compartments.

### LC-MS/MS analysis

#### Instrument and chromatographic conditions

U(H)PLC-MS/MS measurements were performed on a 6460 Triple Quadrupole MS system with a 1290 Infinity LC system equipped with a binary capillary pump G4220A, column oven G1316C, and multisampler G7167B (all Agilent). Quantitative analysis by MS/MS were performed with electrospray ionization (ESI) in MRM (multiple reaction monitoring) mode. Desolvation and nebulization gas was nitrogen. MS/MS data were analyzed with Agilent MassHunter Workstation software version B.07.00. The temperature of the autosampler was 10°C. An Acquity UPLC HSS T3 column (1.8 μm; 100 mm × 2.1 mm) (Waters) was used for separation of the analyte and the internal standard (IS), except for hyperforin and its IS warfarin where an Acquity UPLC CSH Phenyl-Hexyl column (1.7 μm; 50 mm × 2.1 mm) was used. The mobile phase consisted of purified water with 5% MeCN containing 0.1% formic acid (A1) and MeCN containing 0.1% formic acid (B1). Analyses of hypericin and valerenic acid including the IS digoxin were performed on an Acquity UPLC system containing a binary pump, autosampler, and column heater, connected to an Acquity TQD (all Waters). Desolvation and nebulization gas was nitrogen, and collision gas was argon. Flow rate for analysis of all compounds was 0.4 mL/min. The column used was an Acquity UPLC BEH C18 (1.7 μm; 50 mm × 2.1 mm) (Waters). The autosampler temperature was set at 10°C, and the column temperature at 55°C. The mobile phase consisted of purified water containing 0.1% NH_4_OH at pH 10.7 (A2) and MeCN/purified water (9:1) containing 0.1% NH_4_OH (B2). U(H)PLC gradients used for analysis of antipyrine, hyperforin, hypericin, and valerenic acid are given in Supplementary Table S3 (Supplementary Material). MS/MS data were analyzed with MassLynx software version 4.1 (Agilent).

#### Standards and stock solutions

Stock solutions of analytes and IS (Table 1) were prepared as previously described (Spiess et al., 2022a). Details for the calibration curves can be found in Supplementary Figures S8–S11 (Supplementary Material) and in Supplementary Tables S4–S15 (Supplementary Material).

**TABLE 1:** ESI-MS conditions for the analysis of test compounds.

Compound	Range (ng/mL)	MRM transitions	Internal standard	Ionization
Hyperforin	2.5–250	535.38 > 383.2	Warfarin	ESI-
		535.38 > 313.2		
Hypericin	10–1′000	502.8 > 405.1	Digoxin	ESI-
		502.8 > 433.1		
Valerenic acid	10–1′000	232.8 > 41	Digoxin	ESI-
		232.8 > 84		
Antipyrine	5–500	189.1 > 104	Antipyrine-d_3_	ESI+
		189.1 > 56.1		

#### Sample extraction in placenta perfusion medium for antipyrine, hypericin, and valerenic acid

To 200 µL of analyte in PM were added 100 µL of the IS solution, 200 µL of 6% BSA in water, and 800 µL MeCN (1′000 µL for antipyrine). The samples were mixed for 10 min at room temperature on an Eppendorf Thermomixer (1′400 rpm), and finally centrifuged at 10°C for 20 min at 13′200 rpm/16′100 rcf (Centrifuge 5415 R, Eppendorf). An aliquot of 1′100 µL (1′300 µL for antipyrine) supernatant was collected and transferred into a 96-deepwell plate (96-DPW, Biotage) and dried under nitrogen gas flow (Evaporex EVX-96, Apricot Designs). Samples were redissolved with 200 µL of a mixture of A2 and B2 (65:35), followed by 45 min of shaking on an Eppendorf MixMate. Antipyrine was redissolved with 200 µL of A1 and B1 (65:35). Injection was done in full loop mode (2 µL) from the 96-DWP.

#### Sample extraction in placenta perfusion medium for hyperforin

150 μL of the IS in MeOH were added to 50 µL of sample. After centrifugation at 10°C for 20 min at 13′200 rpm/16′100 rcf (Centrifuge 5415R, Eppendorf), 50 µL of supernatant were collected and transferred into HPLC vials. Vials were centrifuged for additional 20 min prior to U(H)PLC-MS/MS analysis in full loop mode (2 µL).

#### Sample extraction in placenta perfusion medium for Transwell samples

For valerenic acid, 150 µL of the IS in MeOH were added to 50 µL of sample, and the mixture centrifuged for 20 min at 13′200 rpm/16′100 rcf at 10°C (Centrifuge 5415R, Eppendorf). An aliquot (50 µL) of the supernatant was collected and transferred into HPLC vials. Vials were centrifuged for additional 20 min prior to U(H)PLC-MS/MS analysis in full loop mode (2 µL).

### Stability assay in placental homogenate

The stability of hyperforin, hypericin, and valerenic acid was assessed in PBS, PM and placental homogenates (prepared according to (Spiess et al., 2022a)) over a period of 360 min, as previously published (Riccardi et al., 2020)). In short, after spiking the various matrices with the study compounds, samples were either processed immediately for U(H)PLC-MS/MS analysis (C0), or kept at 4°C/37°C on an orbital shaker (600 rpm) for 360 min before processing for U(H)PLC-MS/MS analysis. Samples were processed *via* solid phase extraction or protein precipitation prior to analysis.

### Data processing and calculations

**FIGURE 2:**
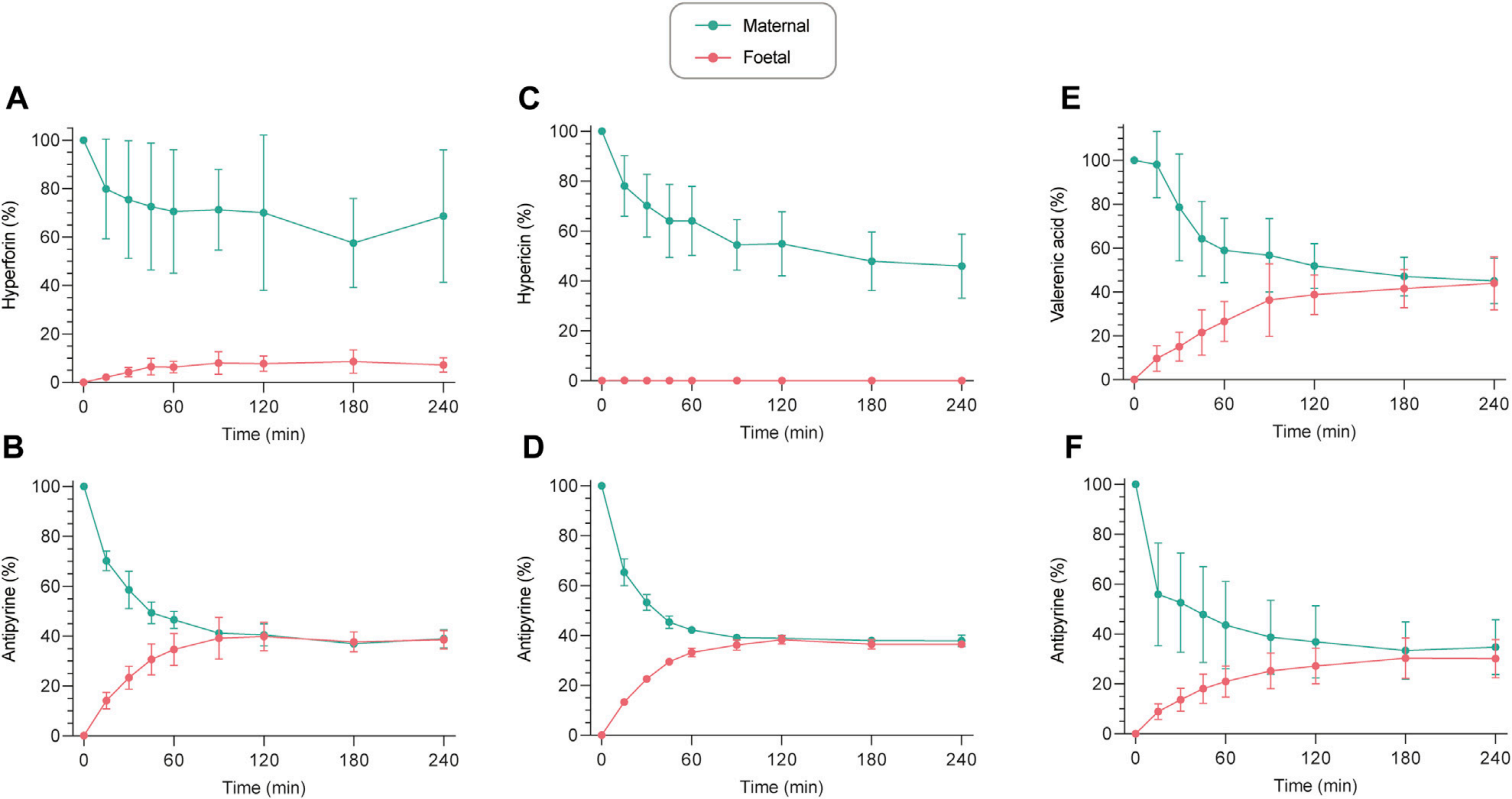
*Ex vivo* human placental perfusion profiles of hyperforin **(A)**, hypericin **(C)**, and valerenic acid **(E)** with corresponding connectivity controls (antipyrine) (**(B,D,F)**, respectively). Concentrations are expressed as a percentage (%) of the initially analyzed concentration in the maternal sample. All values are expressed as mean ± SD of three to four independent experiments. Perfusion profiles with absolute concentrations (ng/mL) can be found in Supplementary Figure S1.

**FIGURE 3:**
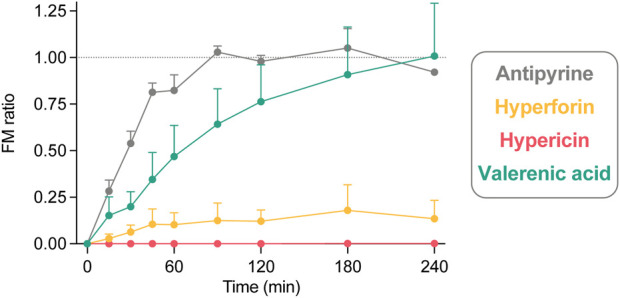
The fetal-maternal concentration ratio (FM ratio; fetal concentration divided by maternal concentration) calculated for each timepoint of hyperforin (n = 4), hypericin (n = 3), and valerenic acid (n = 4), in comparison with antipyrine (n = 3) from control perfusions. The FM ratio is 1.0 when the fetal and maternal concentrations are equal. All data are expressed as mean ± SD.

**FIGURE 4:**
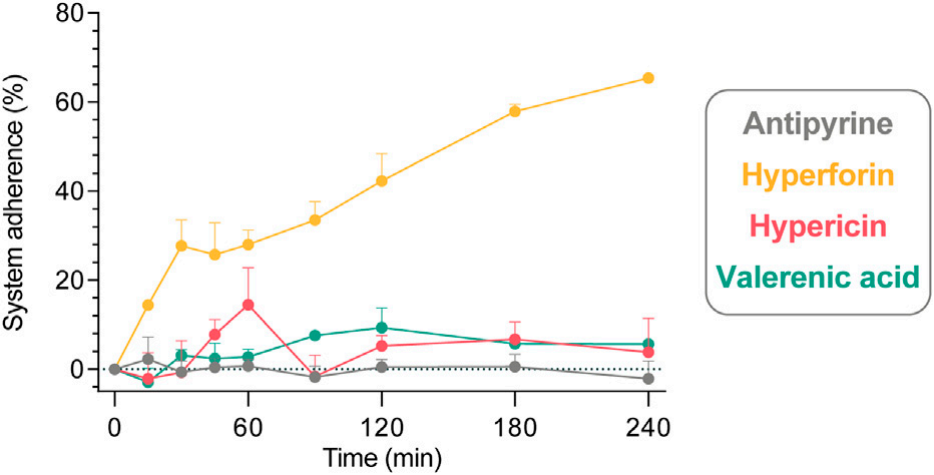
Adherence of study compounds in a 240 min system adherence test (circulation of study compounds through an empty perfusion chamber comprising only the maternal circuit). All compounds were tested individually in three independent experiments, and data are expressed as mean ± SD. Displayed is the percentage (%) of compound (of initially analyzed concentration in the maternal sample) that adheres to the equipment: antipyrine (−2.2% ± 7.0%), hyperforin (65.4% ± 0.7%), hypericin (3.8% ± 13.1%), and valerenic acid (5.6% ± 1.9%) at 240 min.

Concentrations in the placental perfusion profiles (Figure 2) and system adherence tests (Figure 4) were expressed as a percentage (%) of the maternal concentration at the beginning of the perfusion, whereby the maternal concentration, measured in the maternal reservoir, was adjusted to the total volume of the full maternal circuit (maternal reservoir and dead volume of the system). The FM concentration ratio (FM ratio; Figure 3) was calculated for each timepoint and plotted against the perfusion time (min). The final recovery (%) is the sum of the amounts of study compound present in both perfusates at the end of a perfusion, and the sample removed during the experiment. Glucose consumption and lactate production are presented as the sum of changes (from the beginning to the end of perfusion) in total content (μmol) in both circuits, normali*z*ed by total perfusion time (min) and weight (g) of perfused cotyledon. The net release rate of placental hormones – *β*-hCG (U) and leptin (ng) – during the placental perfusion was also normalized by total perfusion time (min) (see (Spiess et al., 2022b) for equations).

### Statistical data analysis

Multiple group comparisons were performed for the glucose consumption, lactate production, *β*-hCG and leptin production using the non-parametric Mann–Whitney U with GraphPad Prism (version 9.3.1 for macOS; GraphPad Software). Probability values of *p* ≤ 0.05 were considered statistically significant. Data are expressed as mean ± SD of three to four independent experiments.

## Results

### *In silico* predictions of physichochemical properties of the test substances

Hyperforin, hypericin and valerenic acid (Figure 1) exhibit rather differing physicochemical properties (Table 2), as determined by the softwares QikProp (Schrodinger LLC) and ACD/Percepta (ACD/Labs release 2020.1.1). Hyperforin and hypericin have both MWs that are markedly higher than that of valerenic acid (and the positive control antipyrine). Hyperforin and hypericin are predicted to be substantially more lipophilic than valerenic acid, which in turn is more lipophilic than the positive control antipyrine, as reflected by the cLogD_pH7.2_ values (Table 2). Hypericin has a markedly higher number of hydrogen bond donors than all other compounds. Finally, the p*K*_a_ values of hyperforin and valerenic acid differ as well. At pH 7.2 of the perfusion experiments, hypericin is present in charge states between 0 and −2, while hyperforin and valerenic acid occur essentially in the single charge state of −1. In contrast, antipyrine is fully uncharged (Table 2).

**TABLE 2:** Predicted physicochemical properties of study compounds.

Compound	Molecular weight (g/mol)	p*K*_a_	No. of hydrogen bond donors	clogD _pH 7.2_	Physiological charge at pH 7.2		
					0 (% of total)	−1 (% of total)	−2 (% of total)
Hyperforin	536.8	4.5	1	7.6	0.2	99.8	0.0
Hypericin	504.4	6.9	6	7.3	30.7	59.3	9.9
Valerenic acid	234.3	4.9	1	2.3	0.5	99.5	0.0
Antipyrine	188.2	No acid p*K*a	0	0.7	100.0	0.0	0.0

### *Ex vivo* characterisation of transplacental transfer

The transplacental transfer of hyperforin, hypericin and valerenic acid resulted in three distinctly different profiles (Figure 2). Hyperforin showed little transfer to the fetal circuit, despite a decrease in the maternal circuit. After 240 min of perfusion, only 7.2% of initial concentration appeared in the fetal compartment, while 68.6% of hyperforin remained on the maternal side (Figure 2A). Hypericin did not cross the human placental barrier within 240 min, while the concentration in the maternal compartment decreased to approximately 46% (Figure 2C). At the same time, antipyrine as a connectivity control reached an equilibrium after 120 min (Figures 2B, D). For valerenic acid, a gradual increase in the fetal compartment and a concomitant decrease in the maternal compartment was observed, reaching an equilibrium after 240 min (44.0% [fetal] vs. 45.0% [maternal] of initial concentration; Figure 2E). The integrity of maternal and fetal circuits was again confirmed with antipyrine (Figure 2F). Perfusion profiles with absolute concentrations can be found in Supplementary Figure S1.

The FM ratio (Figure 3) of hyperforin reached a maximum of 0.18 after 180 min, thereby confirming that only minor amounts crossed the placental barrier. In contrast, the FM ratio of hypericin was zero, as the compound could not be detected in the fetal circuit. For valerenic acid, the FM ratio was 1.01 after 240 min, reflecting the identical concentrations in the fetal and maternal compartments. Antipyrine as a positive control reached an equilibrium between fetal and maternal concentrations after 90 min (FM ratio of 1.03), which remained unchanged during the course of the experiment (FM ratios of 0.98, 1.05, 0.92 at 120, 180, and 240 min, respectively).

### Recovery and stability of the test substances

The system adherence tests (empty perfusions; Figure 4) revealed that negligible proportions of hypericin, valerenic acid and antipyrine were lost over a period of 240 min (calculated values of 3.8%, 5.6%, and −2.2% of initial concentration, respectively). The relative amount of hyperforin which adhered to the perfusion equipment after 240 min was significantly higher (65.4%).

**FIGURE 5:**
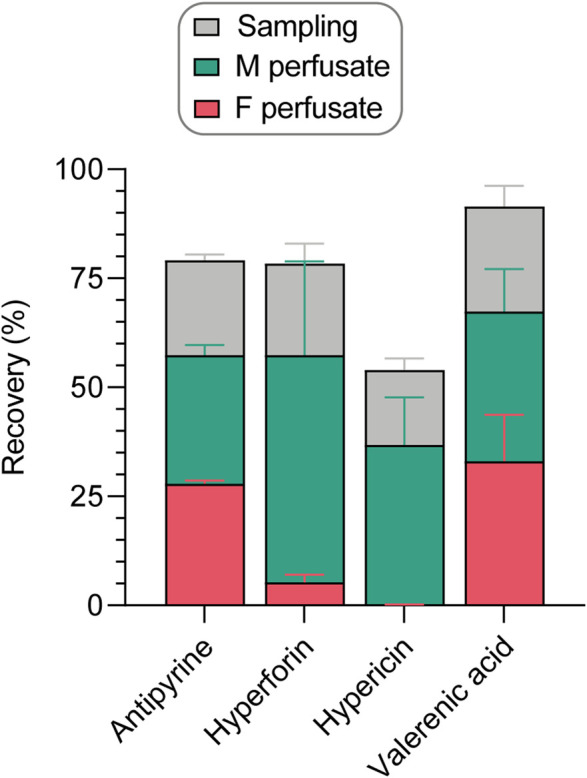
Recovery of study compounds in the human *ex vivo* placental perfusion system, expressed as percentage (%) of amount analyzed in the maternal sample at the beginning of the perfusion. The final recovery was calculated as the sum of compound present in fetal and maternal perfusates at the end of a perfusion, and the amounts sampled during the perfusion from fetal and maternal perfusates (Supplementary Table S1). All data are represented as mean ± SD of three to four independent experiments.

Apart from the system adherence test, several aspects must be considered for the recovery calculations of study compounds during placental perfusions (Figure 5). After 240 min of perfusion, the compounds were distributed in the two compartments (fetal vs. maternal) in the following proportions: antipyrine (27.8% vs. 29.5%), hyperforin (5.3% vs 52.2%), hypericin (0.1% vs. 36.7%), and valerenic acid (33.1% vs. 34.4%). As shown in Supplementary Table S1 and in Figure 5, 17.2%–24.1% of the test compounds were removed by sampling during the perfusion, corresponding to one-fourth of the final recovery. When assessing the final recovery in the placenta perfusions (without considering the results of the independent system adherence test), the following values were obtained: 79.2% ± 2.6% for antipyrine, 78.4% *±* 23.0% for hyperforin, 54.0% *±* 13.7% for hypericin, and 91.5% *±* 19.1% for valerenic acid.

**FIGURE 6:**
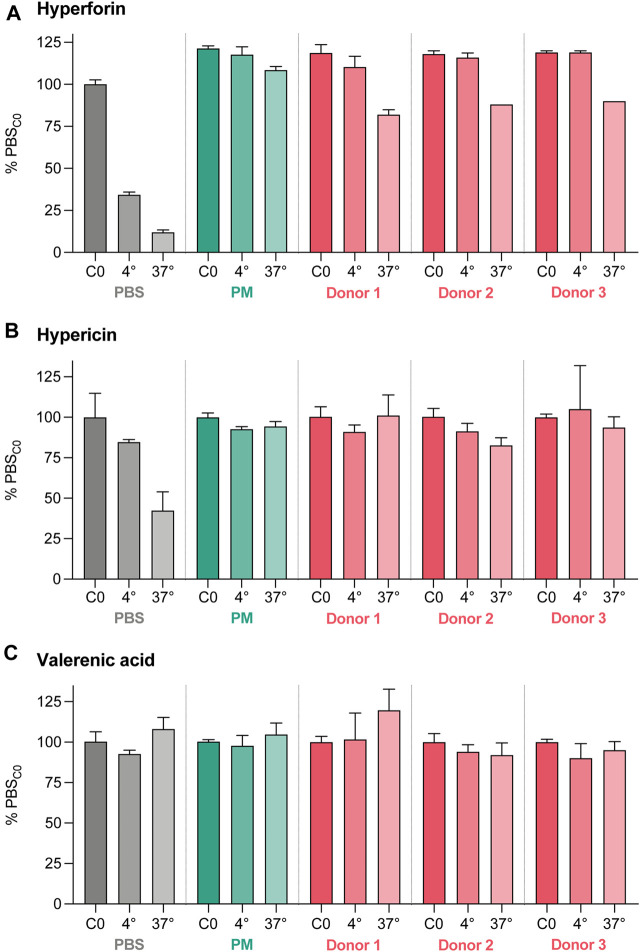
Stability data for hyperforin **(A)**, hypericin **(B)**, and valerenic acid **(C)** expressed as percentage (%) of the initial concentration (C0) in PBS. The stability test was performed for 360 min at two different temperatures (4°C and 37°C) and three different matrices (PBS), perfusion medium (PM), and placental homogenates from three different donors. Differences due to matrix effects were excluded in a separate experiment (Supplementary Figure S2). All data are represented as mean ± SD.

Limited stability of study compounds in the various matrices could lead to misleading results. Therefore, their stability was assessed over 360 min in three different matrices that were relevant for our experiments (PBS, PM, and three placental homogenates [Donors 1–3]) and at two temperatures (4°C, 37°C) (Figure 6). Hyperforin and hypericin were less stable over 360 min at 4°C and 37°C in PBS compared to PM, while the stability data of valerenic acid were very comparable in PBS and PM (approx. 100%). In addition, the solubility of hyperforin was higher in PM than in PBS (121% vs. 100%, Supplementary Figure S2). A loss of hyperforin was observed in the presence of placental homogenate, with 82.0% (Donor 1), 88.0% (Donor 2), and 90.0% (Donor 3) remaining after 360 min. Some degradation in homogenate at 37°C was also observed with hypericin (101.0%, 82.7%, and 93.7% respectively). The use of three different placental homogenates (Donors) resulted in comparable values for all test substances. Some differences attributable to matrix effects could be demonstrated for hyperforin and hypericin in separate experiments (see Supplementary Figure S2).

### Effects on placental function and histology

**FIGURE 7:**
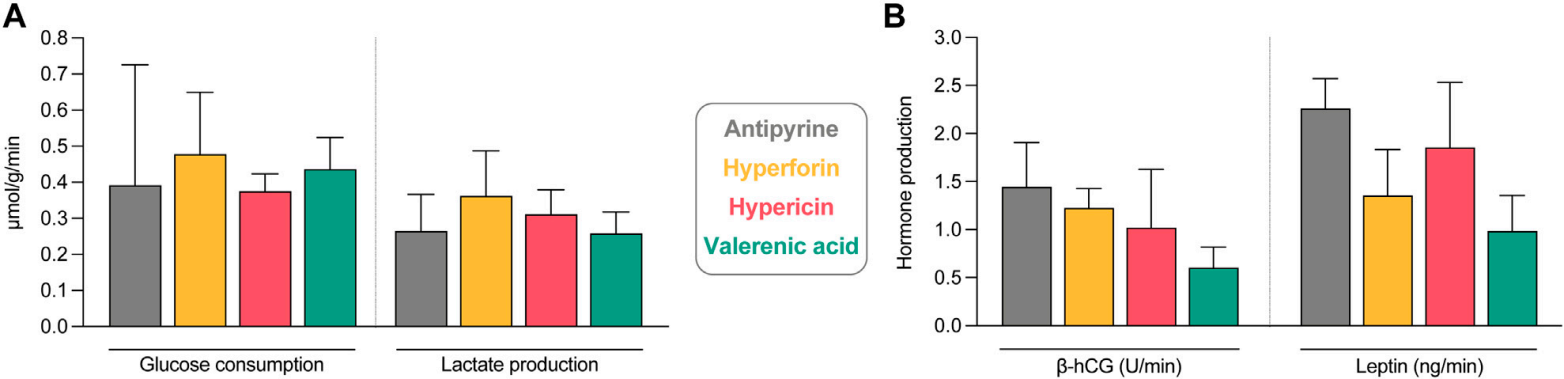
Assessment of tissue viability and functionality during the *ex vivo* human placental perfusion. **(A)** Glucose consumption and lactate production under exposure to study compounds and antipyrine (from control perfusions). Displayed are the changes between beginning and end of the perfusion in fetal and maternal circuits. Data are normalized by the total perfusion time (min) and perfused cotyledon weight (g). All data are represented as mean ± SD of three to four independent experiments. **(B)** Beta-human choriogonadotropin (*β*-hCG) and leptin tissue production of perfusions with study compounds and antipyrine (from control perfusions). Displayed is the net release rate of placental hormones during the placental perfusion, normalized by the total perfusion time (min). All data are represented as mean ± SD of three to four independent experiments (except for the leptin value of antipyrine, where only two values are included). No statistically significant differences were found between the groups (*p* > 0.05 in all cases).

Possible effects of the study compounds on placental metabolic activity and hormonal production were also investigated. The metabolic activity of all perfused placental tissues was similar, as neither glucose consumption nor concomitant lactate production were affected by the study compounds (Figure 7A). With antipyrine (from control perfusions), the total glucose consumption and lactate production during the perfusion were 0.39 and 0.27 µmol/g/min, respectively. Beta-human chorionic gonadotropin (*β*-hCG) and leptin production were determined as an additional measure for placental function and found to be somewhat lower in the presence of all compounds (Figure 7B). However, neither hyperforin, hypericin, nor valerenic acid inhibited their production in a statistically significant manner. This implied that the tissue of all placentae retained their functionality throughout the *ex vivo* perfusion period. A *β*-hCG production of 1.44 U/min and leptin production of 2.26 ng/min was observed in control perfusions with antipyrine only.

Detailed histopathological examination of the perfused tissue revealed that microscopic effects of perfusion were seen in addition to the macroscopically apparent pale tissue (Table 3): i) villous vessels of the perfused side were ≥80% empty (non-perfused area was ≤20% empty of blood), ii) intervillous space of perfused tissue was ≥70% empty of blood (vs. 30%–80% in non-perfused area) and equally or more dilated in contrast to the non-perfused side, iii) formation of hydropic villous changes was found more frequently in the perfused (5%–40%) than in the non-perfused areas (0%–5%), and iv) a clear transition between perfused and non-perfused tissue was observed in most of the cases. The endothelium in the perfused tissue was still viable after 360 min of perfusion. Other histopathological observations that argue against damage to placental tissue after perfusions with the test substances (hyperforin, hypericin, valerenic acid) were i) a low percentage of thrombi in villous vessels (up to 5%) of perfused tissue, ii) no thrombi detectable in vessels of stem villi (perfused and non-perfused), iii) trophoblast vacuolization in perfused areas occurred in a proportion of 0%–30% and was substance-independent, some but not all (two out of four) cotyledons perfused with valerenic acid showed higher proportions (80%–90%), iv) no ruptures of villous vessels, and v) no extravasations into villous stroma. No signs of inflammation were found in any of the examined perfused tissue areas, as neither bacteria nor neutrophils were present in the villous vessels and intervillous spaces. In addition, the assessment of global placental pathology was unremarkable, with no evidence of fetal/maternal vascular malperfusion, villous immaturity, chronic/acute villitis, chronic deciduitis, chorioamnionitis, and bacteria in the non-perfused areas of the placenta.

**TABLE 3:** Detailed histopathological evaluation assessing the microscopic effects of human *ex vivo* placental perfusions with hyperforin (n = 4), hypericin (n = 3) and valerenic acid (n = 4), and the damage of placental tissue in perfused areas compared to non-perfused areas.

		Hyperforin				Hypericin			Valerenic acid			
	Experiment number	1	2	3	4	1	2	3	1	2	3	4
Microscopic effects of perfusion (in %)	Emptiness of villous vessels	95	95	90	80	90	80	85	90	95	80	80
	Emptiness of villous vessels in non-perfused	10	20	5	5	20	5	1	10	10	1	1
	Intervillous spaces without blood	70	95	99	80	90	95	95	95	95	95	95
	Intervillous spaces without blood in non-perfused	30	30	40	60	40	40	80	40	70	50	60
	Dilatated intervillous space	90	90	70	70	60	30	95	70	50	90	90
	Dilatated intervillous space in non-perfused	60	50	70	80	50	30	85	50	20	30	70
	Hydropic changes	20	5	40	10	20	10	5	0	25	40	5
	Hydropic changes in non-perfused	0	0	1	0	5	0	0	0	5	5	0
	Sharp transition perfused/non-perfused	N	Y	Y	N	Y	N	Y	N	Y	Y	Y
Damage of placental tissue (in %)	Thrombi in villous vessels	0	0	0	0	1	0	0	5	1	5	0
	if so: in non-perfused too?	—	—	—	—	ND	—	—	1	ND	1	-
	Vacuolated trophoblast in villi	10	30	10	0	20	20	5	1	90	80	5
	if so: in non-perfused too?	0	0	1	—	0	0	0	ND	0	5	0
Global placental pathology	Else	N	N	N	Intervillous thrombus <5%	CCB	N	CCB	N	Formation of artificial cavities in 10% of perfused areas	N	Singular non-occlusive thrombus in stem villus; CCB

CCB, compensatory core budding; N = no; ND = not determined; Y = yes.

### *In vitro* permeability assays

All results shown so far were obtained with term placentae. To better evaluate the transplacental passage of valerenic acid, the *in vitro* BeWo b30 Transwell model was used. Hyperforin and hypericin were not suited for these experiments as they did not cross the cell-free inserts to a sufficient extent (data not shown). In our hands, a dense BeWo b30 cell layer was obtained 9 days after cell seeding on semi-permeable insert membranes. This timepoint was chosen because i) translocation of NaF (a marker of paracellular passive diffusion) was minimal, with a basolateral amount of 2.8% of the initial NaF concentration (Supplementary Figure S3), ii) TER values reached a value of ≥30 Ωcm^2^ on day 9 after cultivation, which was markedly higher than in previous days (Supplementary Figure S4A), and the C_cl_ was below the expected 5 μF/cm^2^ (Supplementary Figure S4B) and, iii) staining of nuclei (blue) and actin (red) showed gapless growth of BeWo b30 cells on cell culture inserts (Supplementary Figure S5).

**FIGURE 8:**
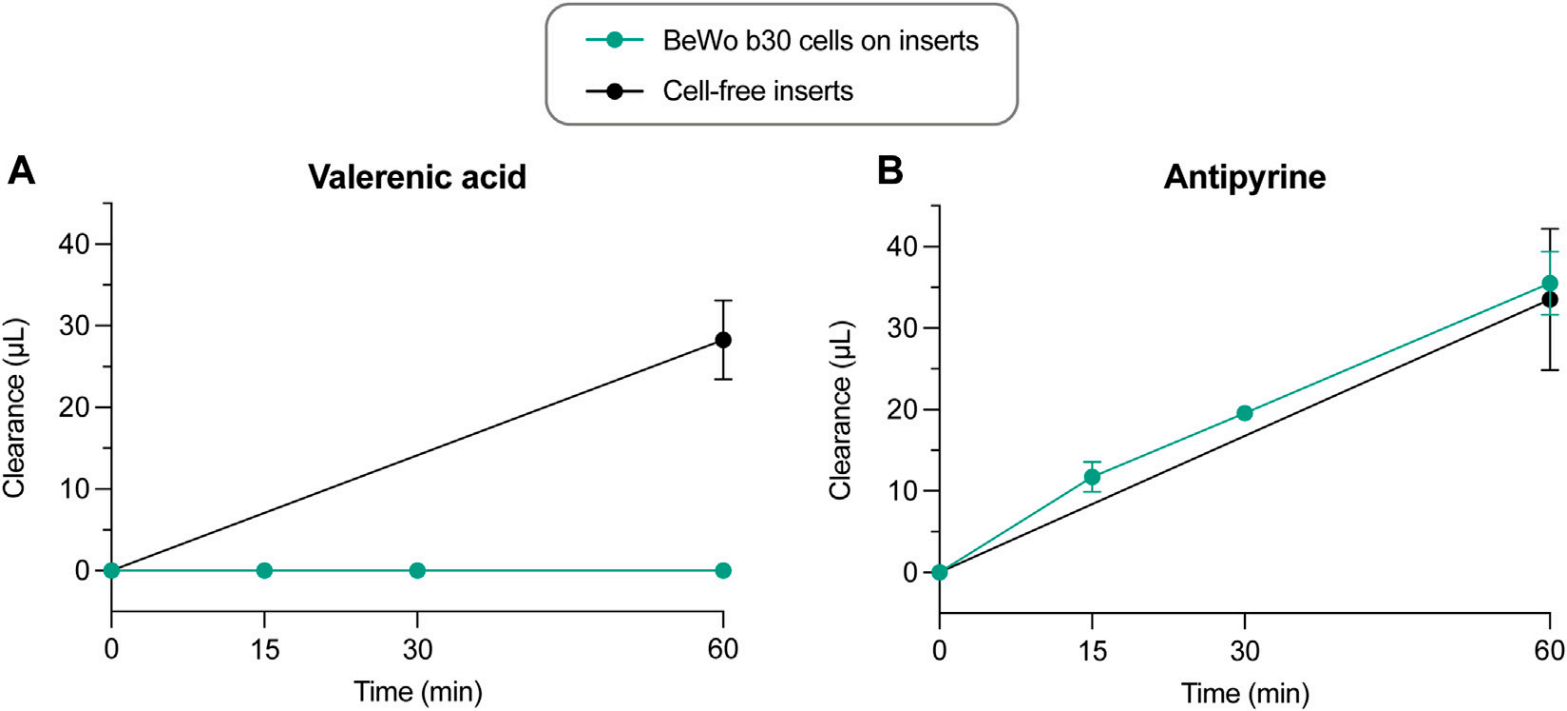
Clearance vs. time curves of control experiments across cell-free inserts (n = 4) and across BeWo b30 cell layers for valerenic acid and antipyrine (n = 3–6). Data are presented as mean ± SD.

In this Transwell model, valerenic acid did not cross the placental cell layer within 60 min to reach detectable concentrations. In contrast, valerenic acid could pass the semi-permeable cell-free insert to the same extent as the positive control antipyrine (Figure 8A; 28.3 μL within 60 min). Antipyrine crossed from the apical to the basolateral compartment through the placental cell layer and the semi-permeable cell-free insert membrane at the same clearance rate (Figure 8B; 35.5 μL and 33.5 μL, respectively). The P_app_ was zero for valerenic acid, and 28.0 × 10^−6^ cm/s for antiyprine. The recoveries after 60 min were 73.7% ± 11.9% for valerenic acid, and 81.9% ± 14.2% for antipyrine, and included the final amounts in the apical, basolateral, and cellular compartment (Supplementary Figure S6). Mean TER and C_cl_ values were similar before and after the permeability experiment with valerenic acid and antipyrine (Supplementary Figure S7).

## Discussion

### Main findings

Our results from the *ex vivo* human term placental perfusion model showed that only minor amounts of hyperforin were transported to the fetal circulation, resulting in a very low FM ratio. Hypericin did not cross the placental barrier, while valerenic acid equilibrated between the maternal and fetal compartments. In addition, metabolic, functional, and histopathological properties of placentae during perfusions were not significantly altered by the test substances. Observations performed with the *in vitro* Transwell model with human placental cells indicated that valerenic acid was unable to cross the cell monolayer, thereby suggesting that the compound may not cross cytotrophoblast layers.

Our observations from the *ex vivo* human term placental perfusion model fit well with the predicted physicochemical properties of the investigated molecules and an expected transport through the placenta barrier by passive diffusion. It is likely that the relatively high MW (>500) of hyperforin and hypericin, together with their ionization states at pH 7.2, hindered their transfer [compare with (Syme et al., 2004; Tetro et al., 2018)]. In contrast, the markedly smaller valerenic acid could equilibrate between maternal and fetal compartments almost as quickly as antipyrine (MWs of 234.3 and 188.2, respectively). Although our results are in line with a transfer by passive diffusion, a possible involvement of additional mechanisms in the transplacental transfer of hyperforin, hypericin and valerenic acid requires further investigation. The results of our stability experiments revealing some degradation of hyperforin, and to a smaller extent hypericin, at 37°C but not at 4°C suggest some metabolization of these compounds through placenta enzymes.

### Strengths and limitations

Plant extracts consist of a variety of different compounds, some of which are present in low amounts only. Therefore, it was crucial to develop sensitive U(H)PLC-MS/MS methods capable of detecting very low concentrations of analytes that one would expect *in vivo* upon oral ingestion of phytomedicines. However, it should be noted that confident statements can only be made within the calibration range (see Materials and methods). A limitation of the study is that concentrations below the limit of quantification had to be assumed to be zero. *Ex vivo* placental perfusion is to date the only experimental model preserving the structural integrity and cell-cell organisation of the organ. It most closely mimics the *in vivo* situation and, therefore, provides good predictions for placental transfer *in vivo*. A disadvantage of the model is that it represents the situation at term, when transplacental transfer is known to be maximal. For compounds that were not transferred in this model (hyperforin and hypericin) one can reasonably assume that they are also not transferred at earlier stages of pregnancy. The *in vitro* Transwell model that mimics the cytotrophoblast monolayer (Bode et al., 2006; Vähäkangas and Myllynen, 2006), provided valuable information for valerenic acid. We opted for not testing hyperforin and hypericin in the Transwell model, since under our experimental conditions these molecules did not cross the membranes of the inserts (in the absence of a cell layer) in a measurable way. The transfer across the membranes was not significantly increased when using high protein concentration in the medium (up to 4% BSA; data not shown). High protein concentrations have been used to improve solubility and, hence, transfer of poorly soluble compounds (Füller et al., 2018). Similar limitations of such permeability experiments with hypericin have been previously described in the Caco cell model (Verjee et al., 2015). A limitation common to both models is that they cannot fully represent the *in vivo* situation, as they do not take into account aspects such as dissolution, absorption, distribution, metabolism, and excretion of the compounds (Poulsen et al., 2009; Hutson et al., 2011; Myllynen and Vahakangas, 2013), which strongly influence transplacental transfer. Finally, the two methods only allow a study of short-term toxic effects on placental tissue and cells, while in pregnancy the placentae are exposed for extended periods to the substances. Especially with compounds that accumulate in cell membranes, such as hypericin [(Verjee et al., 2015); own unpublished observations], this might lead to an underestimation of possible undesired effects.

### Recovery of hyperforin, hypericin and valerenic acid

Experiments with hyperforin and hypericin required special attention, due to their high lipophilicity and poor solubility. In the absence of biological material, hyperforin showed a significant loss in the empty perfusions (65.4% over a 4-h period) which could be due to adsorption to tubing/equipment or precipitation. The higher recovery in the presence of placental tissue could be explained by the higher protein content in the system. The presence of protein is needed to stabilize and solubilize this compound, as described in literature (Füller et al., 2018), and this was reflected by the high stability in the PM but not in presence of PBS (Figure 6). The amounts in the fetal and maternal compartments, and the amounts removed by sampling were comparable for hyperforin (78.4%) and for antipyrine (79.2%). In addition, loss of hyperforin in the incubations with placental homogenates may indicate a possible metabolisation. The lowest final recovery was found for hypericin (54.0%), although there was only a small loss due to system adsorption (3.8% over a 4-h period). Again, stability was good in PM but not in PBS (Figure 6). However, the recovery data did not take into account the percentage in placental tissue. Interestingly, fluorescence microscopic images showed a considerable accumulation of hypericin in placental cells (data not shown), which is similar to previous observations with Caco-2 cells (Verjee et al., 2015). In the placental perfusion model valerenic acid showed good stability and high recovery, thereby facilitating data interpretation.

### St. John’s wort: Comparison with previous *in vitro* studies

Previous *in vitro* studies showed that extracts from St. John’s wort had no negative impact on placental cells at concentrations up to 30 µg/mL (cytotoxicity, apoptosis) or 100 µg/mL (genotoxicity, metabolic activity, and influence on placental cell differentiation) (Spiess et al., 2021). Our present data with term placentae suggest that a possible fetal exposure to hypericin and hyperforin is likely minimal. This is particularly important, as transplacental transport is maximal at term due to a decrease of cell layer thickness and number of cell layers towards the end of pregnancy (van der Aa et al., 1998; Vähäkangas and Myllynen, 2006). *In vitro*, hyperforin showed no effects on viability, metabolic activity, and on induction of placental cell differentiation at concentrations up to 30 μM. However, hyperforin led to increased apoptosis and genotoxic effects starting at concentrations of 3 and 10 μM, respectively, and inhibited FSK-induced placental cell differentiation at concentrations of ≥1 µM (Spiess et al., 2022b). It should be noted that these test concentrations were significantly higher than reported plasma concentrations in humans. Upon oral administration of a single dose of 300 mg St. John’s wort extract containing 14.8 mg hyperforin a maximum plasma concentration of 150 ng/mL (approx. 0.28 µM) was reached (Biber et al., 1998). C_max_ values of 83.5 ng/mL (≙ 0.16 µM) and 122 ng/mL (≙ 0.23 µM) hyperforin were determined after single dose administration of 612 mg and 900 mg dry extract, respectively (Schulz et al., 2005b; a). However, the hyperforin content in commercially available products can vary considerably (Schäfer et al., 2019). Given that low amounts of hyperforin can possibly cross the transplacental barrier, and our recent data on inhibition of cell differentiation at ≥ 1 µM concentrations in BeWo cells (Spiess et al., 2022b), it may be prudent to resort in pregnancy to products with a low hyperforin content. Hypericin lowered the viability of placental cells already at 1 μM concentrations, and apoptotic and genotoxic effects were seen at concentrations of 1 and 10 μM, respectively (Spiess et al., 2022b). The amount of hypericin in commercially available drugs varies. For products marketed in Switzerland and Germany, amounts ranging from 0.08 mg to 0.21 mg per 100 mg of tablet have been reported (Schäfer et al., 2019). In human volunteers, plasma concentrations of 2.2 ng/mL hypericin (≙ 4.36 nM) have been found (Jackson et al., 2014). The plasma levels of hypericin thus are significantly lower than the concentrations found toxic in BeWo cells (Spiess et al., 2022b). However, plasma levels of hypericin may increase upon co-administration of certain other drugs (Jackson et al., 2014).

### Valerian: Comparison with previous *in vitro* studies

At concentrations up to 30 µg/mL, no signs of cytotoxicity or apoptosis, and at concentrations up to 100 µg/mL, no signs of genotoxicity, alteration of metabolic activity and placental cell differentiation were observed in BeWo cells for the valerian extract (Spiess et al., 2021). Valerenic acid was not permeable in the *in vitro* Transwell model mimicking continuous cytotrophoblast layers that are likely to play a role in the placenta barrier at early stages of pregnancy. However, it reached an equilibrium between the fetal and the maternal circulation in the *ex vivo* placental perfusion model representative for late stages of pregnancy. With respect to valerenic acid, concentrations up to 30 μM did not lower viability of BeWo b30 cells, and no increase in apoptosis or genotoxicity, and no negative effect on the metabolic activity and cell differentiation were observed (Spiess et al., 2022b). Valerenic acid contents ranging from 1.21 mg/g to 2.46 mg/g product have been reported in products marketed in Australia (Shohet et al., 2001), while 0.57 mg–2.20 mg valerenic acid per tablet/capsule have been found in products marketed in Switzerland (Winker et al., manuscript under review). For valerenic acid, maximal plasma concentrations of 2.3 ng/mL (≙ 9.82 nM) to 3.3 ng/mL (≙ 14.08 nM) have been reported (Anderson et al., 2005; Anderson et al., 2010). Considering that valerenic acid did not affect cell viability in BeWo b30 cells at concentrations up to 30 μM (Spiess et al., 2022b), the valerenic acid content in products, and reported plasma concentrations, there appears to be a large safety margin.

### Final statement

Hyperforin could only cross the complex placental barrier to a very small extent, while hypericin appeared to be non-permeable. Valerenic acid crossed the placental barrier at term when permeability is higher, but not in the *in vitro* BeWo transfer model representative of cytotrophoblast monolayer. Taken together, our data suggest that when treating mild mental disorders with St. John’s wort and valerian extracts, fetal exposure to hypericin and hyperforin and, at early stages of pregnancy to valerenic acid, is likely to be low. Our study included so far only single compounds that are considered as relevant for the pharmacological properties of St. John’s wort and valerian. Given that the entire extracts, and not just single compounds, are considered as the active ingredient of phytomedicines, the possible influence of the extract matrix on placental permeability of these compounds should be evaluated. Recent *in vitro* data in BeWo cells with St. John’s wort and valerian extracts, and with hyperforin, hypericin and valerenic acid (Spiess et al., 2021; Spiess et al., 2022b) suggest moreover no toxicity at concentrations to be expected in humans at the recommended extract doses, but caution against using products containing high amounts of hyperforin.

## Data Availability

The original contributions presented in the study are included in the article/Supplementary Material, further inquiries can be directed to the corresponding authors.

## Abbreviations

BSA, bovine serum albumin; C_cl_, electrical capacitance; DMSO, dimethyl sulfoxide; 96-DWP, 96-deepwell plate; EMA, European Medicine Agency; ESI, electrospray ionization; FM, fetal-maternal; FM ratio, fetal-maternal concentration ratio; HMPC, Committee on Herbal Medicinal Products; *β*-hCG, beta-human chorionic gonadotropin; IS, internal standard; MeCN, acetonitrile; MRM, multiple reaction monitoring; MW, molecular weight; NaF, sodium fluorescein; P_app_, apparent permeability coefficient; PM, perfusion medium; SSRIs, selective serotonin reuptake inhibitors; TER, transepithelial electrical resistance.
