# Supplementary material for: Transplacental passage of hyperforin, hypericin, and valerenic acid
Source: Front Pharmacol. 2023 Mar 31;14:1123194. doi: 10.3389/fphar.2023.1123194 (PMC10103840; doi:10.3389/fphar.2023.1123194)
Supplement: Supplementary file 1 [file DataSheet1.PDF]

Supplementary Material

Transplacental passage of hyperforin, hypericin, and valerenic acid

Deborah Spiess[1,2§], Vanessa Fabienne Abegg[2§], Antoine Chauveau[2], Joshua Rath[1], Andrea Treyer[2], Michael Reinehr[3], Sabrina Kuoni[1], Mouhssin Oufir[2#], Olivier Potterat[2], Matthias Hamburger[2*], Ana Paula Simões-Wüst[1*]

[1] Department of Obstetrics, University Hospital Zurich, University of Zurich, Zurich, Switzerland

[2] Division of Pharmaceutical Biology, Department of Pharmaceutical Sciences, University of Basel, Basel, Switzerland

[3] Department of Pathology and Molecular Pathology, University Hospital Zurich, Zurich, Switzerland

[#] Present address: Oncodesign SA, Villebon-sur-Yvette, France

[§] These authors contributed equally to the work

*** Correspondence:**

Ana Paula Simões-Wüst, anapaula.simoes-wuest@usz.ch

Matthias Hamburger, matthias.hamburger@unibas.ch

1 Supplementary Figures and Tables

1.1 Supplementary Figures

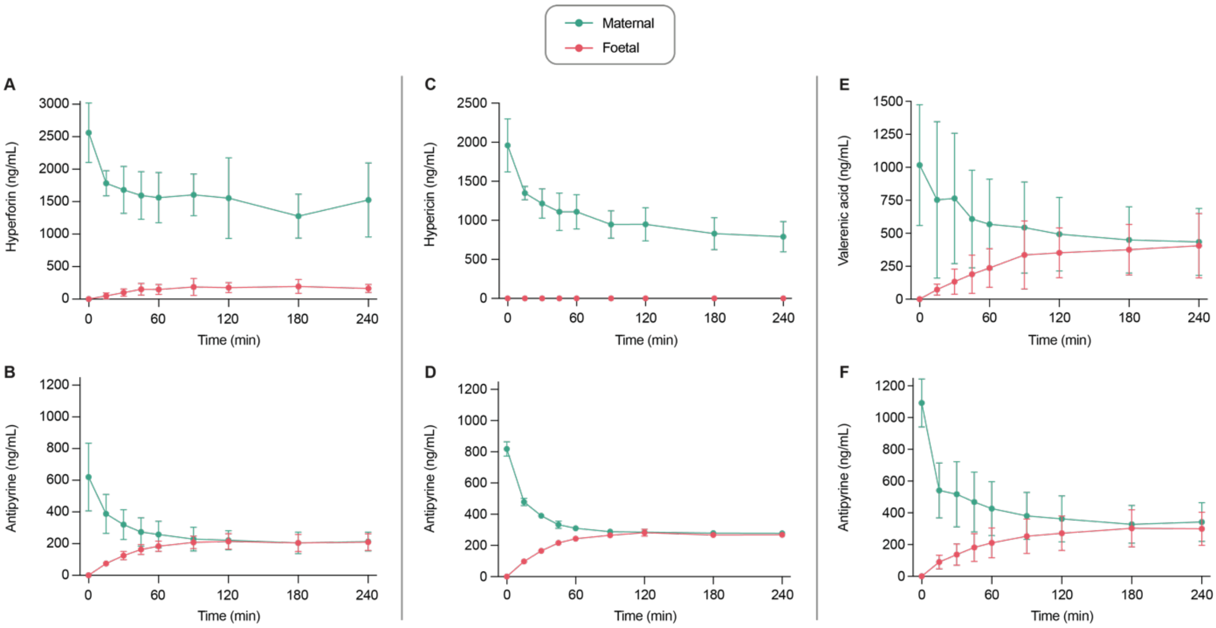

Supplementary Figure 1. *Ex vivo* human placental perfusion profiles of hyperforin (A), hypericin (C) and valerenic acid (E) with corresponding connectivity control (antipyrine) transfers (B, D and F, respectively). Concentrations are expressed as absolute concentrations in ng/mL (● maternal and ● foetal). All values are expressed as mean ± SD of three to four independent experiments.

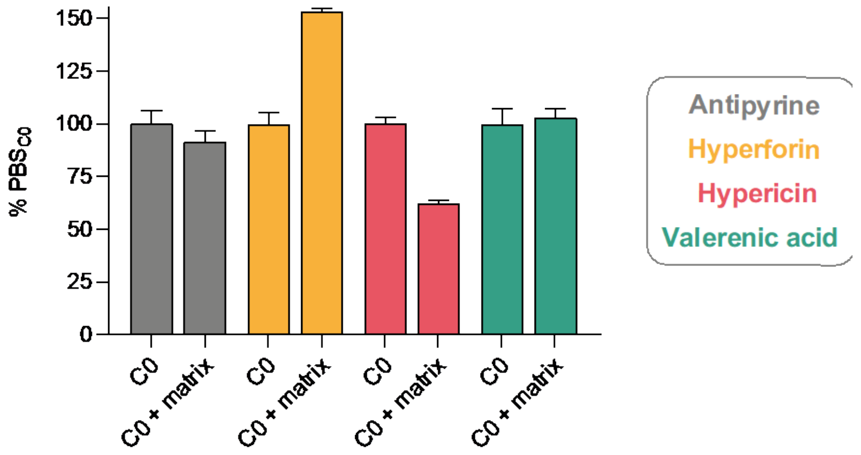

Supplementary Figure 2. Matrix effects by the homogenate on the substances antipyrine, hyperforin, hypericin, and valerenic acid. The compounds were spiked into PBS and then diluted with either an equal volume of PBS (C0) or an equal volume of tissue homogenate (C0 + matrix). The samples were processed for LC-MS analysis within 1-3 min after the dilution to avoid interference of any stability issues. All values are expressed as mean ± SD of three to four independent experiments.

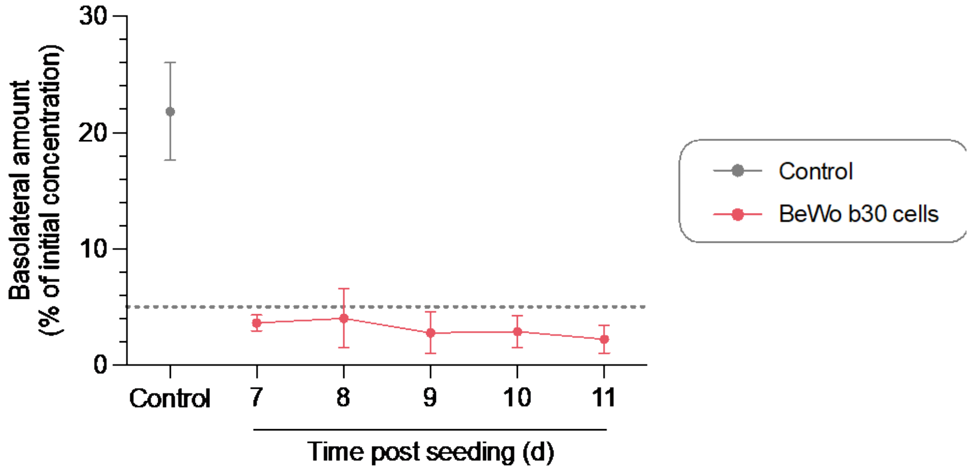

Supplementary Figure 3. Evaluation of monolayer formation using a sodium fluorescein (NaF) exclusion assay. The y-axis displays the amount of NaF in the basolateral compartment as percent (%) of initial concentration (5 µM) after 60 min. Control consists of a cell-free insert only (n=3). Data with cells represents the mean ± standard deviation of 3 biologically independent experiment with three to four technical replicates.

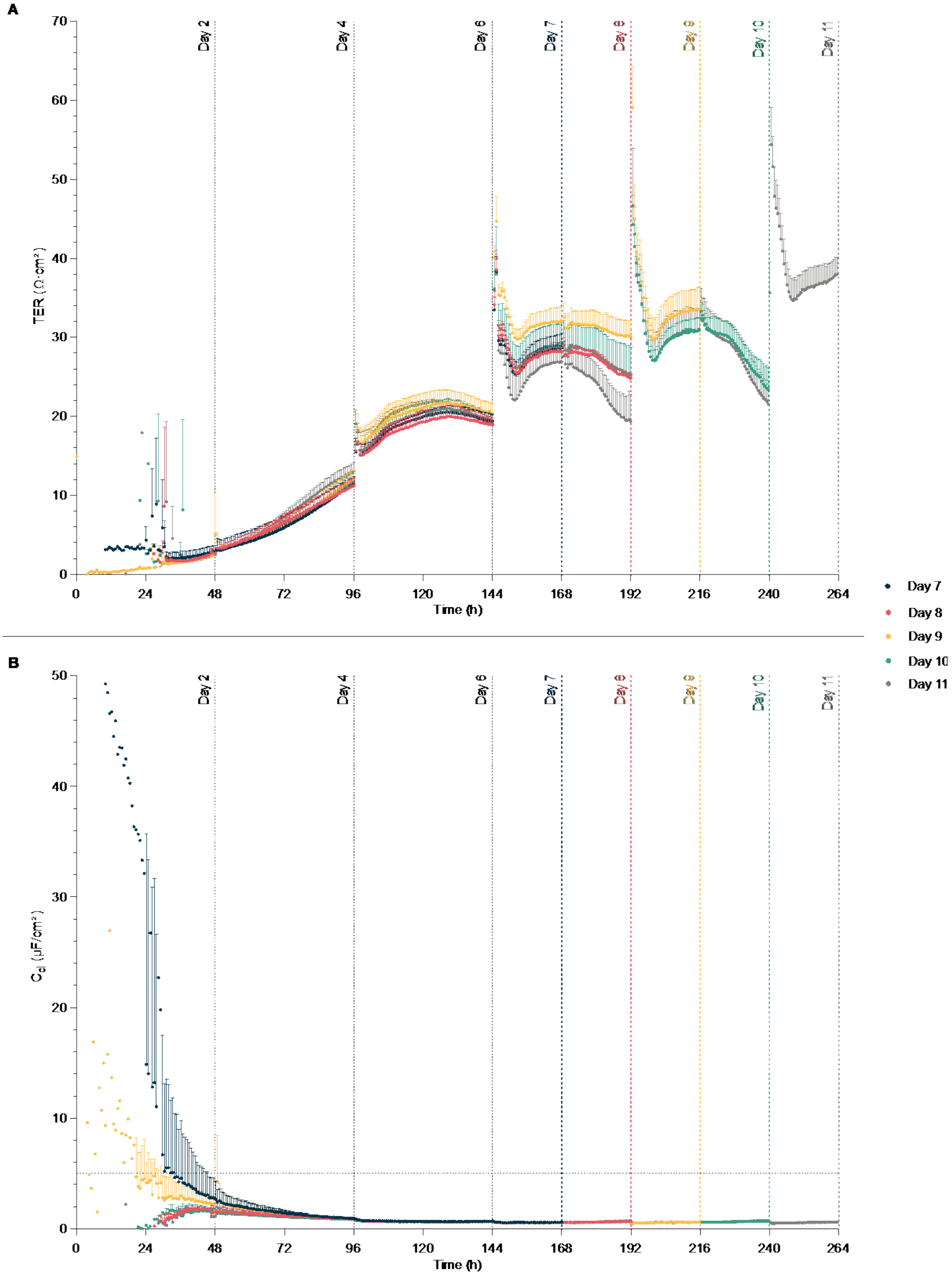

Supplementary Figure 4. Transepithelial electrical resistance (TER, A) and electrical capacitance C_{cl} (B) values evaluating the monolayer formation of BeWo b30 cells grown on Transwell® polycarbonate membrane insets. Cell culture medium was changed every other day (days 2, 4, 6, 8, and 10, respectively). Each day includes data (mean ± SD) from a biologically independent experiment with four technical replicates, with four inserts removed daily (starting from day 7) to perform a sodium fluorescein (NaF) exclusion assay.

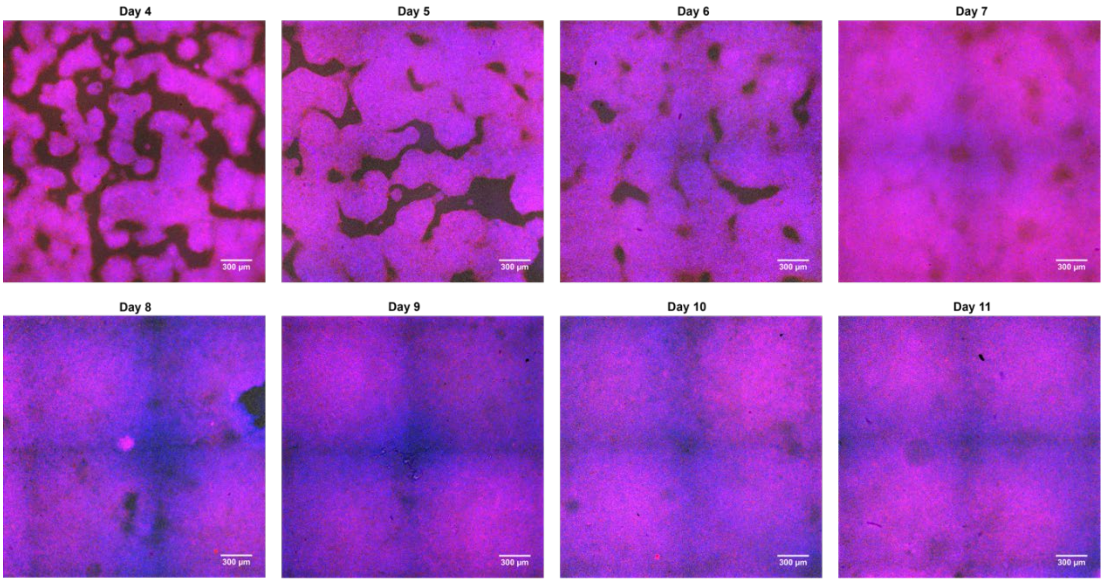

Supplementary Figure 5. Staining of nuclei (blue) and actin (red) of BeWo b30 cells growing on cell culture inserts after 4-11 days of cultivation. The images are representative of two independent cultures. A total of 49 individual tiles were acquired and automatically stitched to an overall image of the membrane, with only a selection of four tiles of the center shown here.

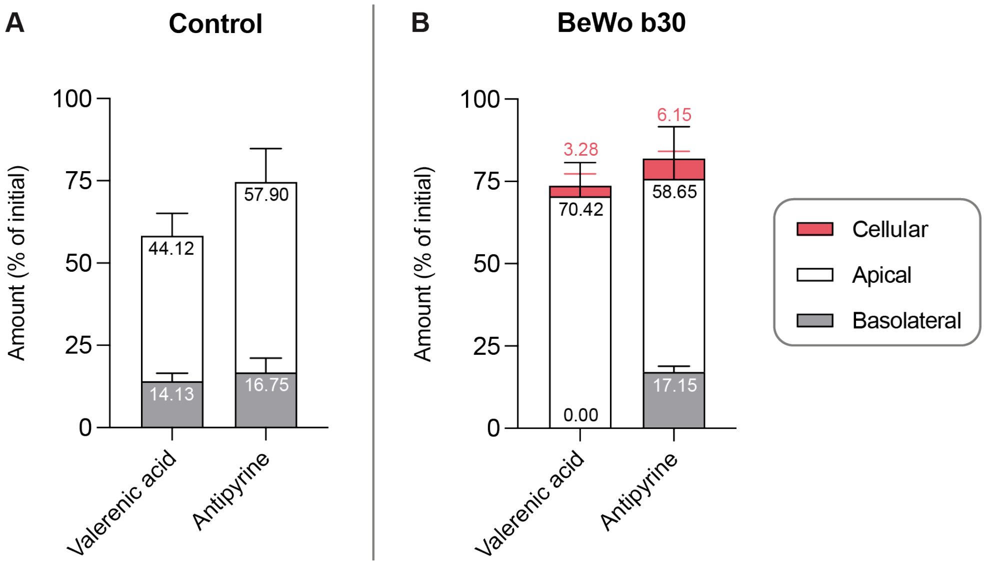

Supplementary Figure 6. Recoveries of valerenic acid and antipyrine using cell-free inserts (control, A) and BeWo b30 cells on inserts (B). The y-axis displays the amount of valerenic acid or antipyrine in the apical, basolateral, or cellular compartment as percent (%) of initial concentration (5 µM) after 60 min. Control consists of a cell-free insert only (n=4), which was pre-incubated with cell culture medium for 9 days to match the experimental conditions of the Transwell experiments with BeWo b30 cells (n=3-6). Data with cells represents the mean ± standard deviation of three biologically independent experiment with three technical replicates.

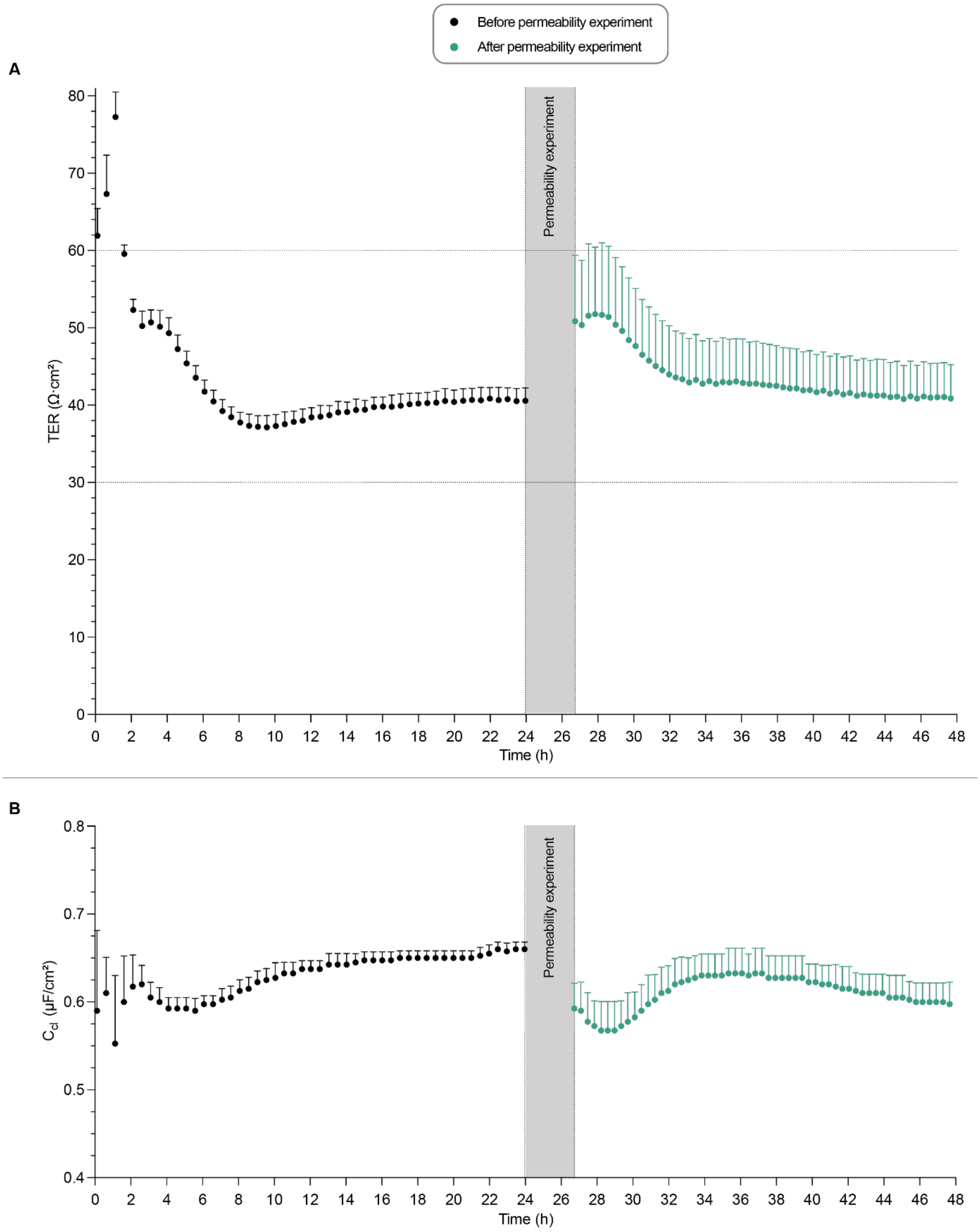

Supplementary Figure 7. Development of transepithelial electrical resistance (TER, A) and electrical capacitance C_{cl} (B) values. Measurements started on day 8 of cultivation and simultaneously 24 h prior to the BeWo b30 exposure to valerenic acid and antipyrine. The measurement was paused during the permeability experiment (day 9 of cultivation) and recorded for another 24 h after exposure. Each data set is representative of a biologically independent experiment with four technical replicates.

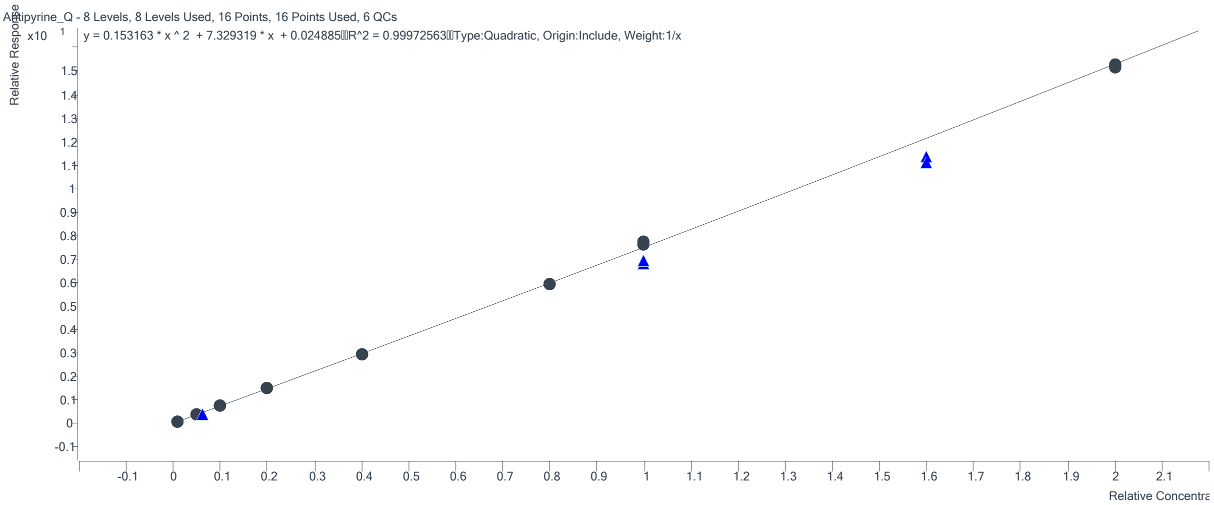

Supplementary Figure 8. Calibration curve of antipyrine (calibrators are shown as circles, quality controls [QCs] are shown as triangles).

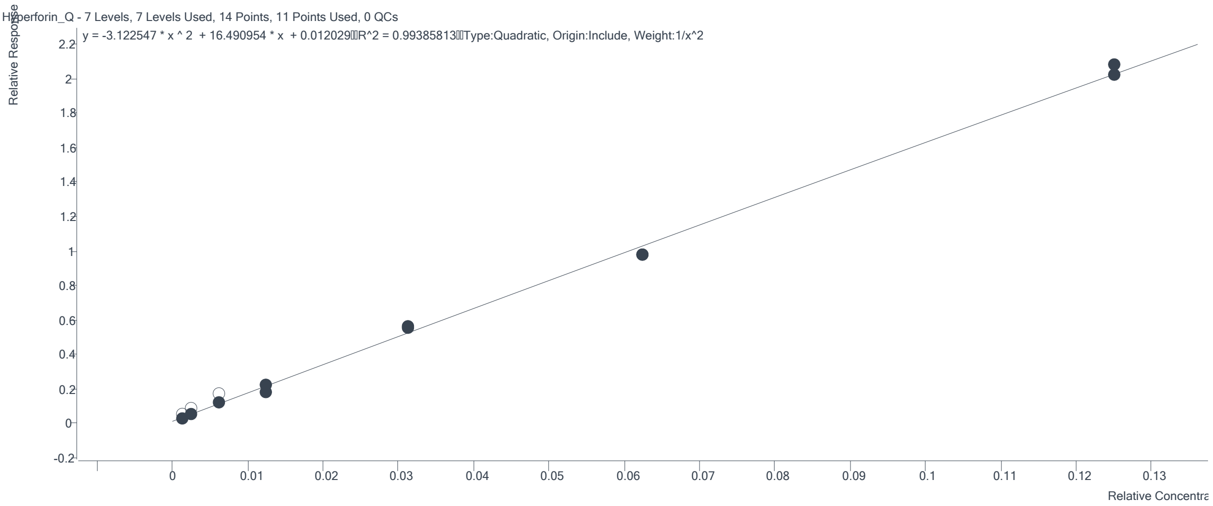

Supplementary Figure 9. Calibration curve of hyperforin (calibrators are shown as circles where open circles are excluded values and closed circles are included values).

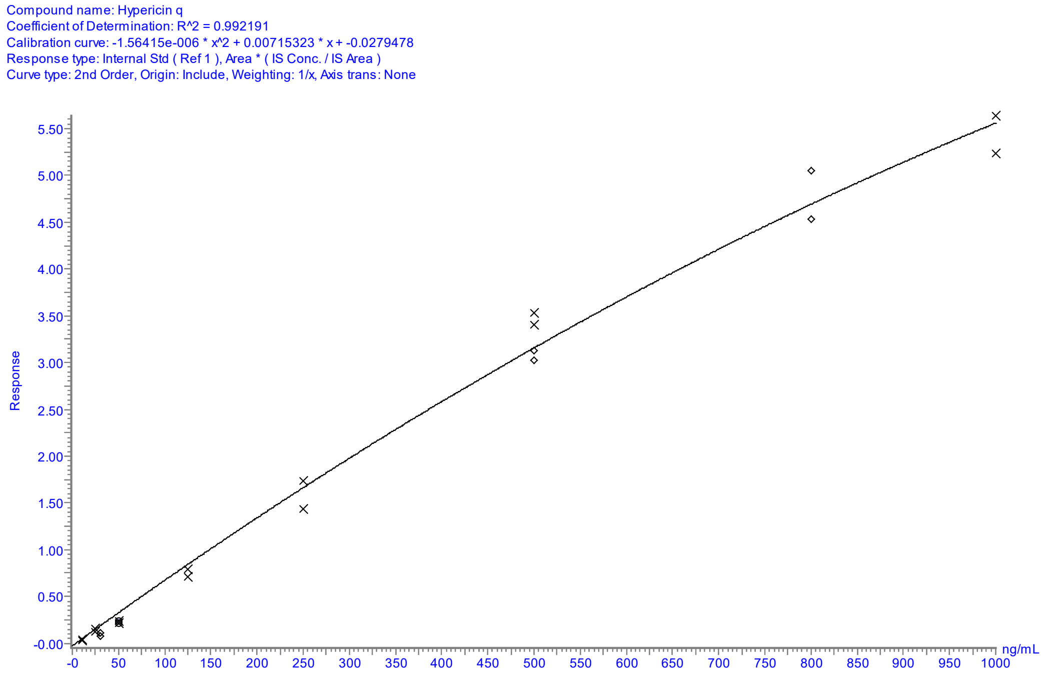

Supplementary Figure 10. Calibration curve of hypericin (calibrators are shown as x and quality controls [QCs] are shown as diamonds).

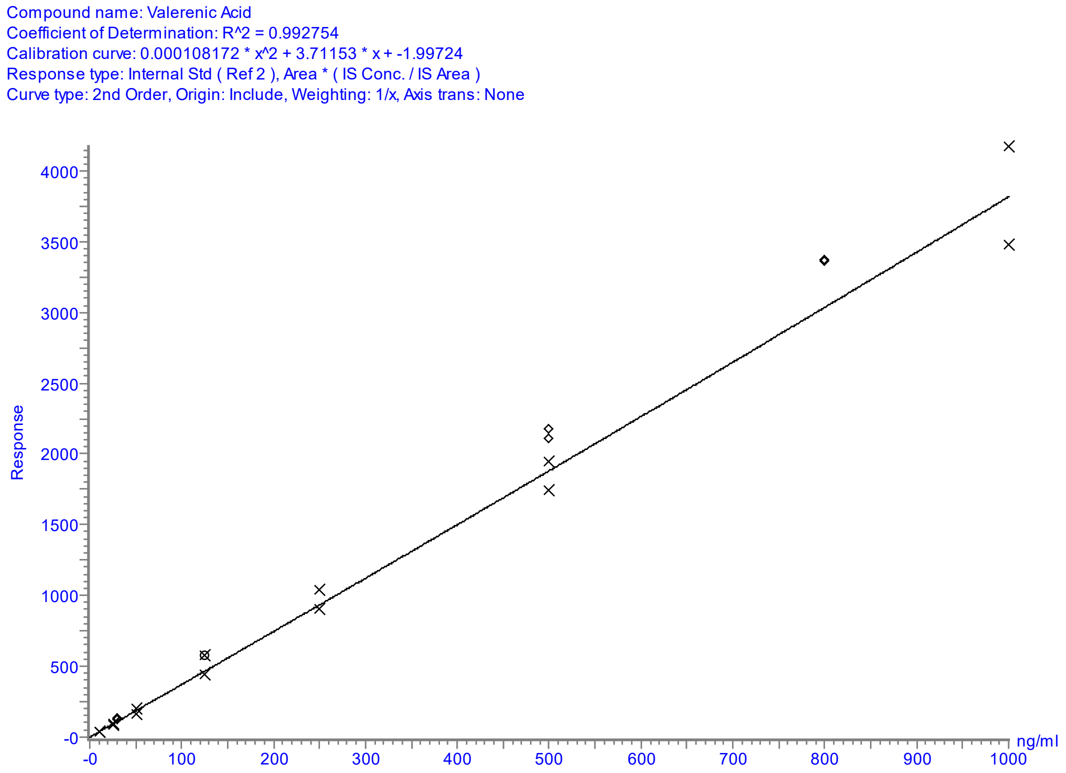

Supplementary Figure 11. Calibration curve of valerenic acid (calibrators are shown as x and quality controls [QCs] are shown as diamonds).

1.2 Supplementary Tables

Supplementary Table 1. Recovery of study compounds after 240 min of *ex vivo* human placental perfusion in relative amounts (% found) ± SD in different compartments.

	M perfusate (% found)	F perfusate (% found)	Sampling (% found)	Final recovery* (% found)
Antipyrine	29.5 ± 2.4	27.8 ± 0.8	21.8 ± 1.3	79.2 ± 2.6
Hyperforin	52.2 ± 21.5	5.3 ± 1.8	21.0 ± 4.5	78.4 ± 23.0
Hypericin	36.7 ± 11.0	0.1 ± 0.1	17.2 ± 2.7	54.0 ± 13.7
Valerenic acid	34.4 ± 9.8	33.1 ± 10.7	24.1 ± 4.8	91.5 ± 19.1

M = maternal; F = foetal; * sum of compound present in foetal and maternal perfusates at the end of a perfusion and the amount sampled during the perfusion (sampling from foetal and maternal perfusates).

Supplementary Table 2. Characteristics of placentae used, and data from individual perfusions with all study compounds (hyperforin, n=4; hypericin, n=3; and valerenic acid, n=4).

	Hyperforin				Hypericin			Valerenic acid			
Experiment number	1	2	3	4	1	2	3	1	2	3	4
Placenta weight (g)	570	470	730	520	515	630	490	460	570	460	550
Cotyledon weight (g)	17.21	7.93	6.68	17.38	14.48	14.97	21.26	10.87	6.69	27.08	13.26
Volume loss* (M, mL)	2	6	-2	2	0	-4	-3	3	9	-3	0
Volume loss* (F, mL)	6	16	8	1	15	13	8	0	9	16	5
Preparation time° (min)	30	19	15	14	18	16	20	14	16	17	14
Cannulation time§ (min)	30	14	24	25	13	15	18	36	ND	18	23
Open pre-phase (min)	20	20	20	20	20	20	20	20	20	20	20
Close pre-phase (min)	20	20	20	20	20	25	20	20	20	20	21
Perfusion time (min)	240	240	240	240	240	240	240	240	240	240	240

* Total volume loss at the end of the perfusion time; M = maternal; F = foetal; ° time from birth to begin of cannulation; § time from cannulation to begin of open pre-phase; ND = not determined.

Supplementary Table 3. U(H)PLC gradients and flow rate for antipyrine, hyperforin, hypericin, and valerenic acid. For antipyrine and hyperforin, the mobile phase consisted of A1 (purified water with 0.5% MeCN and 0.1% formic acid) and B1 (MeCN with 0.1% formic acid). For hypericin and valerenic acid, the mobile phase consisted of A2 (purified water with 0.1% NH_4OH at pH 10.7) and B2 (MeCN/purified water with 0.1% NH_4OH, ratio 9:1).

**Antipyrine**				
	**Time (min)**	**A1 (%)**	**B1 (%)**	**Flow rate (mL/min)**
	0.00	100.0	0.0	
	0.50	100.0	0.0	
	3.00	37.89	62.11	
	3.01	0.0	100.0	0.4
	4.00	0.0	100.0	
	4.01	100.0	0.0	
	5.00	100.0	0.0	
**Hyperforin**				
	**Time (min)**	**A1 (%)**	**B1 (%)**	**Flow rate (mL/min)**
	0.00	100.0	0.0	
	1.00	100.0	0.0	
	2.00	0.0	100.0	0.4
	3.80	0.0	100.0	
	3.81	100.0	0.0	
	5.00	100.0	0.0	
**Hypericin**				
	**Time (min)**	**A2 (%)**	**B2 (%)**	**Flow rate (mL/min)**
	0.00	95.0	5.0	0.4
	1.00	95.0	5.0	

	6.00	30.0	70.0	
	6.10	0.0	100.0	
	7.00	0.0	100.0	
	7.10	95.0	5.0	
	8.00	95.0	5.0	
**Valerenic acid**				
	**Time (min)**	**A2 (%)**	**B2 (%)**	**Flow rate (mL/min)**
	0.00	95.0	5.0	
	1.00	95.0	5.0	
	6.00	30.0	70.0	
	6.01	0.0	100.0	0.4
	7.00	0.0	100.0	
	8.00	0.0	100.0	

Supplementary Table 4. Calibrators and calibration curve parameters for the determination of antipyrine.

Response: $A \times Conc^2 + B \times Conc + C$, 1/X weighting, Quadratic regression, included origin (n=12).

	Concentration (ng/mL)								Regression parameters			
	**5**	**25**	**50**	**100**	**200**	**400**	**500**	**1000**	**A**	**B**	**C**	**R^2**
Mean	5.236	24.358	50.440	101.986	199.923	393.158	508.523	979.559	6.98E-01	8.090	0.054	0.999
SD	0.446	0.584	2.912	5.947	8.119	8.690	20.043	63.632	1.71E+00			
CV%	8.521	2.396	5.772	5.831	4.061	2.210	3.941	6.496				
RE%	4.730	-2.568	0.879	1.986	-0.039	-1.711	1.705	-2.044				

Supplementary Table 5. Calibrators and calibration curve parameters for the determination of hyperforin.

Response: $A \times Conc^2 + B \times Conc + C$, 1/$X^2$ weighting, Quadratic regression, included origin (n=6).

	Concentration (ng/mL)							Regression parameters			
	**2.5**	**5**	**12.5**	**25**	**62.5**	**125**	**250**	**A**	**B**	**C**	**R2**
Mean	2.424	5.283	12.631	25.229	62.450	117.365	256.008	1.18E+01	11.013	0.006	0.989
SD	0.094	0.151	1.131	2.692	4.936	2.031	17.714	1.34E+01			
CV%	3.861	2.862	8.958	10.671	7.904	1.730	6.919				
RE%	-3.033	5.657	1.045	0.917	-0.081	-6.108	2.403				

Supplementary Table 6. Calibrators and calibration curve parameters for the determination of hypericin.

Response: $A \times Conc^2 + B \times Conc + C$, 1/X weighting, Quadratic regression, included origin (n=6).

	**Concentration (ng/mL)**							**Regression parameters**			
	**10**	**25**	**50**	**100**	**250**	**500**	**1000**	**A**	**B**	**C**	**R^2**
Mean	10.217	24.550	47.825	121.633	249.400	513.250	992.567	-7.49E-06	0.744	0.874	0.994
SD	1.105	2.270	3.530	9.308	18.055	48.067	71.287	1.15E-05			
CV%	10.819	9.248	7.382	7.652	7.239	9.365	7.182				
RE%	2.167	22.750	-4.350	21.633	-0.240	2.650	-0.743				

Supplementary Table 7. Calibrators and calibration curve parameters for the determination of valerenic acid.

Response: $A \times Conc^2 + B \times Conc + C$, 1/X weighting, Quadratic regression, included origin (n=8).

	**Concentration (ng/mL)**							**Regression parameters**			
	**10**	**25**	**50**	**100**	**250**	**500**	**1000**	**A**	**B**	**C**	**R^2**
Mean	10.150	23.967	50.129	122.657	255.588	496.313	1000.343	8.02E-05	2.331	-0.881	0.993
SD	0.695	1.433	4.555	11.075	27.297	43.344	58.347	1.81E-04			
CV%	6.847	5.981	9.087	9.029	10.680	8.733	5.833				
RE%	1.500	19.833	0.257	22.657	2.235	-0.738	0.034				

Supplementary Table 8. Quality control samples of antipyrine at low (QCL), medium (QCM), and high (QCH) levels (n=12).

	**QCL**	**QCM**	**QCH**
	**30**	**500**	**800**
Mean	27.592	497.772	827.402
SD	0.735	28.970	59.383
CV%	2.663	5.820	7.177
RE%	-8.027	-0.446	3.425

Supplementary Table 9. Quality control samples of hyperforin (n=2).

	QCL<br>7.5	QCM<br>125	QCH<br>200
Mean	7.092	139.810	210.736
SD	-	4.071	12.660
CV%	-	2.912	6.007
RE%	-5.444	11.848	5.368

Supplementary Table 10. Quality control samples of hypericin (n=6).

	QCL<br>30	QCM<br>500	QCH<br>800
Mean	30.967	487.783	818.800
SD	3.493	15.674	55.443
CV%	11.281	3.213	6.771
RE%	3.222	-2.443	2.350

Supplementary Table 11. Quality control samples of valerenic acid (n=8).

	QCL<br>30	QCM<br>500	QCH<br>800
Mean	30.100	495.171	803.738
SD	2.435	34.844	62.814
CV%	8.089	7.037	7.815
RE%	0.333	-0.966	0.467

Supplementary Table 12. Carry-over assessment for antipyrine (see Materials and Methods, LC-MS/MS analysis (Spiess et al., 2022); LLOQ = lower limit of quantification; IS = internal standard).

		Peak area (cts)				Individual carry-over (%)		Mean carry-over (%)	
		Blank sample		LLOQ					
**Run**	**Replicate**	**Analyte**	**IS**	**Analyte**	**IS**	**Analyte**	**IS**	**Analyte**	**IS**
1	1	178.51	393.50	8873.33	102852.16	2.01	0.38	1.20	0.39
	2	28.99	345.72	7304.05	85516.75	0.40	0.40		
2	1	698.64	52.30	9365.15	85714.84	7.46	0.06	6.94	0.24
	2	636.10	390.00	9908.07	91407.39	6.42	0.43		
3	1	3515.76	301.60	10991.17	110887.04	31.99	0.27	19.79	0.18
	2	729.93	92.20	9610.16	109394.35	7.60	0.08		
4	1	755.22	87.74	10547.34	94071.40	7.16	0.09	11.60	0.33
	2	7840.21	729.76	48904.81	130372.79	16.03	0.56		
5	1	1617.47	93.09	12237.63	117057.68	13.22	0.08	17.78	0.13
	2	2851.55	214.78	12761.11	117699.58	22.35	0.18		
6	1	8209.80	174.88	29676.75	102812.32	27.66	0.17	-	0.22
	2	5990.02	172.57	14188.00*	63909.47	-	0.27		
							**Mean carry over**	8.19	0.16

*Analyte outside acceptance criteria, carry over not calculated

Supplementary Table 13. Carry-over assessment for hyperforin.

Run	Replicate	Peak area (cts)				Individual carry-over (%)		Mean carry-over (%)	
		Blank sample		LLOQ					
		Analyte	IS	Analyte	IS	Analyte	IS	Analyte	IS
1	1	200.80	38.89	2999.13	93244.19	6.70	0.04	-	-
	2	382.24	34.22	8107.83*	154775.14*	-	-		
2	1	243.15	117.53	1226.37	86029.54	19.83	0.14	22.05	0.13
	2	515.77	204.69	2125.54	157231.96	24.27	0.13		
3	1	118.44	242.55	1226.37	86029.54	9.66	0.28	7.08	0.16
	2	109.48	77.16	2437.34	188650.41	4.50	0.04		
							**Mean carry over**	14.56	0.15

*Analyte outside acceptance criteria, carry over not calculated

Supplementary Table 14. Carry-over assessment for hypericin.

		Peak area (cts)				Individual carry-over (%)		Mean carry-over (%)	
Run	Replicate	Blank sample		LLOQ					
		Analyte	IS	Analyte	IS	Analyte	IS	Analyte	IS
1	1	1.07	0.00	29.10	649.46	3.68	0.00	2.82	0.00
	2	0.45	0.00	22.99	666.85	1.95	0.00		
2	1	0.00	0.00	28.57	-	0.00	-	5.07	#DIV/0!
	2	5.51	0.00	54.37	-	10.13	-		
3	1	21.01	0.22	91.60	2066.26	22.94	0.01	23.04	0.01
	2	20.22	0.00	87.37	2286.41	23.14	0.00		
							**Mean carry over**	14.05	0.00

Supplementary Table 15. Carry-over assessment for valerenic acid.

		Peak area (cts)				Individual carry-over (%)		Mean carry-over (%)	
Run	Replicate	Blank sample		LLOQ					
		Analyte	IS	Analyte	IS	Analyte	IS	Analyte	IS
1	1	0.00	0.00	13.02*	1481.91*	-	-	-	-
	2	0.00	0.00	34.05	1888.26	0.00	0.00		
2	1	0.00	0.00	33.51	1415.78	0.00	0.00	0.00	0.00
	2	0.00	0.00	35.89	1565.70	0.00	0.00		
3	1	0.00	1.48	37.76	1102.88	0.00	0.13	0.00	0.07
	2	0.00	0.00	32.23	1382.67	0.00	0.00		
4	1	0.15	0.00	29.41	751.35	0.50	0.00	-	-
	2	0.00	0.00	15.34*	631.10*	-	-		
							**Mean carry over**	0.00	0.03

*Analyte outside acceptance criteria, carry over not calculated

Reference

Spiess, D., Abegg, V.F., Chaveau, A., Treyer, A., Oufir, M., Reinehr, M., Duong, E., Potterat, O., Hamburger, M., and Simões-Wüst, A.P. (2022). Placental passage of protopine in an ex vivo human perfusion system. *Planta Med*. doi: 10.1055/a-1829-9546
